# Ampicillin promotes the biofilm formation of *Shewanella putrefaciens* through the c-di-GMP-regulated BpfAGD system

**DOI:** 10.1128/spectrum.02906-25

**Published:** 2025-11-19

**Authors:** Rui Shi, Di Sun, Jiawen Liu, Jing Yang, Jingrong Zhu, Cong Liu, Weijie Liu

**Affiliations:** 1 Department of Microbiology, School of Life Sciences, Jiangsu Normal University 216827, Xuzhou, Jiangsu, China; Reichman University, Herzeliya, Israel

**Keywords:** *Shewanella*, biofilm, β-lactam antibiotics, c-di-GMP, BpfAGD system

## Abstract

Although many *Shewanella* strains are naturally resistant to some β-lactam antibiotics, research into the effect of these antibiotics on *Shewanella* biofilm formation is scarce. *Shewanella putrefaciens* is not only known as an important seafood spoilage bacterium but can also cause infection in several aquatic animals. In addition, it is a rare opportunistic human pathogen. The present study found that although some antibiotics from cephalosporins, carbapenems, and monobactams repress the biofilm formation of *S. putrefaciens* CN32, multiple penicillin antibiotics increase its biofilm formation. Further experiments showed that ampicillin can increase intracellular c-di-GMP levels by regulating 16 DGCs/PDEs. This increases biofilm formation of *S. putrefaciens* CN32 by controlling the BpfAGD system. These penicillin antibiotics were also found to increase biofilm formation by *Shewanella oneidensis* MR-1. In addition, the biofilm formation by *S. oneidensis* MR-1 was increased by carbapenem antibiotics but repressed by cephalosporins and monobactam antibiotics. This study provides a theoretical foundation for future research into the impact of β-lactam antibiotics on the biofilm formation of *Shewanella*, as well as the mechanisms that regulate this process.

**IMPORTANCE:**

The resistance of bacteria in biofilms to antibacterial agents is much higher than that of planktonic bacteria. Bacterial antibiotic resistance in biofilms and bacterial biofilm formation induced by certain antibiotics are now key concerns. Many *Shewanella* strains are naturally resistant to some β-lactam antibiotics. However, research into whether β-lactam antibiotics induce *Shewanella* biofilm formation is scarce. This study examined the impact of various β-lactam antibiotics on the biofilm formation of *Shewanella putrefaciens* CN32, as well as the mechanism by which ampicillin promotes biofilm formation. This provides guidance on the correct use of antibiotics and improves our understanding of the molecular mechanisms underlying bacterial resistance and antibiotic-induced biofilm formation. This could lay theoretical groundwork for controlling biofilms in the future.

## INTRODUCTION

Biofilms are structural communities of sessile microbial cells embedded in a self-produced extracellular polymeric substance, which are mainly composed of various components such as extracellular polysaccharides, proteins, and eDNA (1–4). These extracellular matrixes protect bacterial cells from adverse external environments, thereby increasing their resistance to various forms of stress (3). In nature, most microorganisms can form biofilms under certain conditions (1). Biofilm formation has an enormous impact on industrial production and human health (5, 6). For pathogenic bacteria, biofilm formation is the major cause of chronic bacterial infections and drug resistance (6). Antibacterial agent resistance of bacteria in biofilms is 10–1,000 times higher than that of planktonic bacteria (7, 8). Now, bacterial antibiotic resistance in biofilms is a key concern, as is bacterial biofilm formation that is induced by certain antibiotics (9). Some antibiotics, when present at concentrations below the minimum inhibitory concentration (sub-MIC), allow susceptible strains to continue growing, but they can also cause bacteria to form biofilms (10). Once antibiotics induce biofilm formation, biofilm formation increases bacterial resistance to antibiotics. Antibiotics induce biofilm formation in two manners: one is that antibiotics act as signal molecules to promote biofilm formation directly (11, 12); the other is that antibiotics regulate other physiological processes, thereby inducing biofilm formation (13, 14). Thus, research is needed to establish whether different antibiotics trigger the biofilm formation by different bacteria and to understand how this process is initiated.

The biofilm development includes four stages: initial attachment, microcolony formation, biofilm maturation, and biofilm dispersal (15). c-di-GMP is a critical second messenger that regulates bacterial biofilm development (16). The widely accepted regulatory model is that low intracellular c-di-GMP levels are associated with a planktonic lifestyle, whereas high intracellular c-di-GMP levels tend to promote biofilm formation (16, 17). The synthesis of the c-di-GMP is catalyzed by diguanylate cyclase (DGC) containing GGDEF domain, whereas the degradation of the c-di-GMP is catalyzed by phosphodiesterase (PDE) containing EAL or HD-GYP domain (16, 17). Some DGCs/PDEs have signal-sensing domains at their N-terminus, which can regulate c-di-GMP synthesis or degradation in response to specific intracellular or extracellular signals (18, 19).

β-Lactam antibiotics are widely used antimicrobial agents that disrupt cell wall synthesis, leading to bacterial cell lysis and death (20, 21). Depending on the characteristics of their unique β-lactam ring structure, β-lactam antibiotics are classified as penicillins, cephalosporins, carbapenems, and monobactams (22). β-Lactam antibiotics can induce multiple bacteria to form biofilms, such as *Haemophilus influenzae* and *Staphylococcus aureus* (14, 23). However, the induction mechanisms differ between bacteria. Thus, research into how β-lactam antibiotics induce biofilm formation will provide a theoretical basis for their future scientific use.

*Shewanella* are Gram-negative bacteria, an aquatic environmental microorganism belonging to the γ-proteobacteria, which are widespread in a variety of environments due to their respiratory and physiological diversity and their ability to thrive at low temperatures (24, 25). The versatility of *Shewanella* respiration allows them to utilize a wide range of electron acceptors, which enables them to play a crucial role not only in bioremediation and bioengineering applications but also in the geochemical cycling of iron, manganese, nitrogen, and carbon (25–27). *Shewanella putrefaciens* is not only known as an important seafood spoilage bacterium (28) but can also cause infection in several aquatic animals (29–31). In addition, *S. putrefaciens* is a rare opportunistic human pathogen (32). Previous studies have reported that many *Shewanella* strains are naturally resistant to some β-lactam antibiotics (33, 34). However, research into whether β-lactam antibiotics induce *Shewanella* biofilm formation is rare.

The biofilm formation of several *Shewanella* species is significantly regulated by the BpfAGD system (35–38). BpfA is an outer-membrane adhesion protein, which regulates cell surface localization and cell-cell adhesion (35, 36, 39). BpfG is a periplasmic protease and BpfD is an inner-membrane-spanning c-di-GMP effector (35, 36). BpfAGD system regulates biofilm formation in response to intracellular c-di-GMP levels (35, 36). High intracellular c-di-GMP levels promote the formation of the c-di-GMP-BpfD complex (35, 36). This complex interacts with BpfG, sequestering it on the inner membrane (35, 36). This results in BpfA being located on the outer membrane, thereby promoting biofilm formation (35, 36). Low levels of intracellular c-di-GMP prevent BpfD from interacting with BpfG (35, 36). This allows BpfG to process and release BpfA from the cell surface, resulting in planktonic growth (35, 36).

This study examined the impact of various β-lactam antibiotics on the biofilm formation of *S. putrefaciens* CN32. The results revealed that six penicillin antibiotics increased biofilm formation; however, the three other classes—cephalosporins, carbapenems, and monobactams—repress biofilm formation by *S. putrefaciens* CN32. Ampicillin was used to investigate how penicillin antibiotics regulate biofilm formation. Previous studies showed that there are 47 c-di-GMP metabolic enzymes in *S. putrefaciens* CN32 (36, 40), including 19 potential DGCs containing GGDEF domain, 10 potential PDEs containing EAL and HD-GYP domains, and 18 potential DGCs or PDEs containing GGDEF-EAL dual domain (Table S1). The results showed that ampicillin can increase intracellular c-di-GMP levels by regulating 16 DGCs/PDEs. This increases biofilm formation of *S. putrefaciens* CN32 by controlling the BpfAGD system. To determine the effect of β-lactam antibiotics on biofilm formation of other *Shewanella*, *S. oneidensis* MR-1 was used. The results showed that six penicillin antibiotics and two carbapenem antibiotics increased the biofilm formation of *S. oneidensis* MR-1. Thus, multiple penicillin antibiotics increase biofilm formation by *S. putrefaciens* CN32 and *S. oneidensis* MR-1. In contrast, cephalosporins and monobactam antibiotics repress biofilm formation by both bacteria.

## RESULTS

### Penicillin antibiotics promote biofilm formation of *S. putrefaciens* CN32

**Fig 1:**
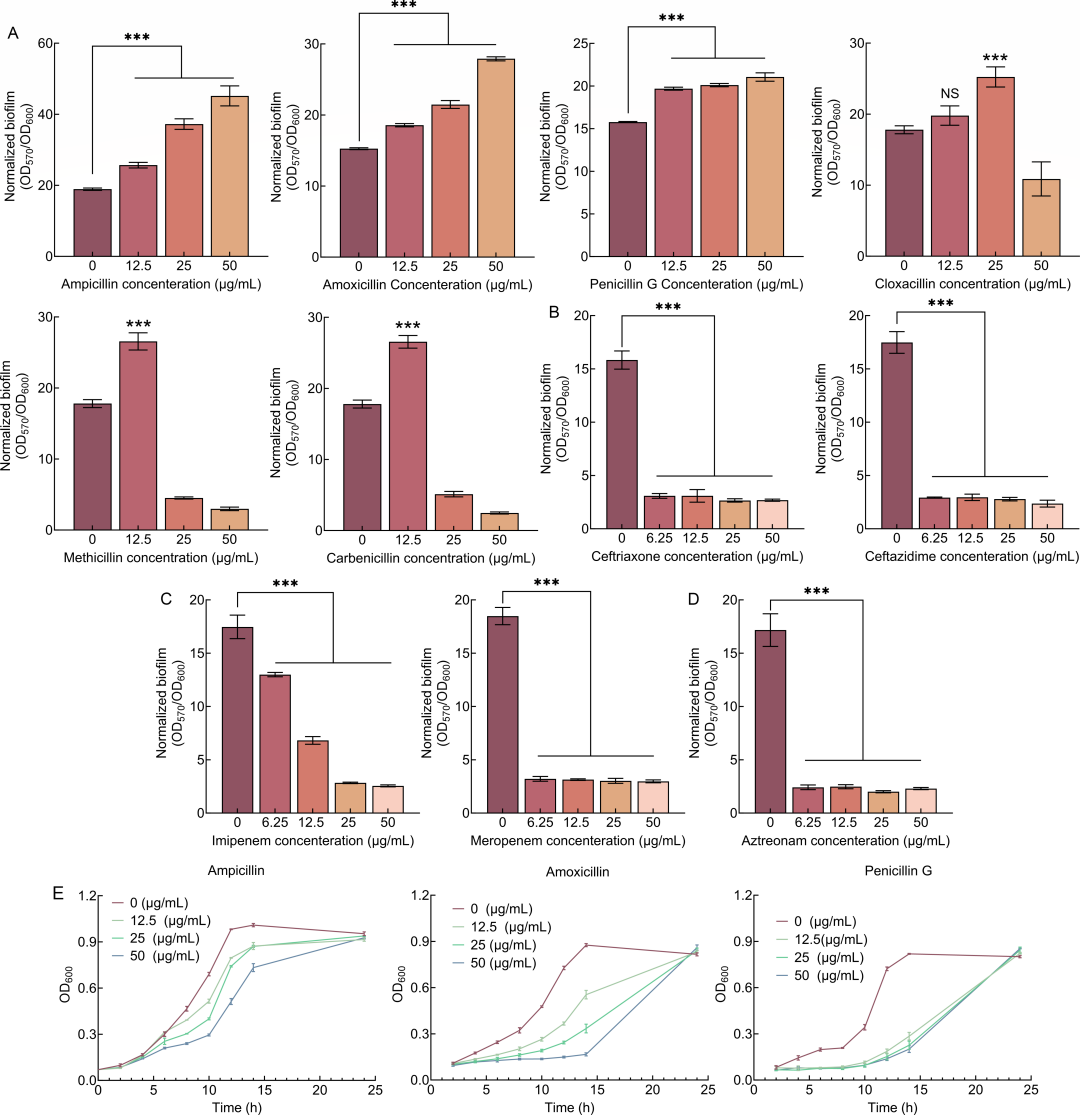
The effect of multiple β-lactam antibiotics on biofilm formation of *S. putrefaciens* CN32. (**A**) The effect of six penicillin antibiotics on biofilm formation at 24 h. (**B**) The effect of two cephalosporin antibiotics on biofilm formation at 24 h. (**C**) The effect of two carbapenem antibiotics on biofilm formation at 24 h. (**D**) The effect of one monobactam antibiotic on biofilm formation at 24 h. (**E**) The effect of different concentrations of ampicillin, amoxicillin, and penicillin G on planktonic cell growth. Two-sided Student’s *t*-test was used to analyze the statistical significance (NS: no significance; ***P < 0.001).

The effects of commonly used β-lactam antibiotics from four different classes on the biofilm formation of *S. putrefaciens* CN32 were determined. The results showed that all used penicillin antibiotics can increase the normalized biofilm biomass (the ratio of biofilm biomass to cell growth, OD_570_/OD_600_) of *S. putrefaciens* CN32, although the required antibiotic concentration varies (Fig. 1A). The other three classes of antibiotics all repress the normalized biofilm biomass of *S. putrefaciens* CN32 (Fig. 1B through D).

**Fig 2:**
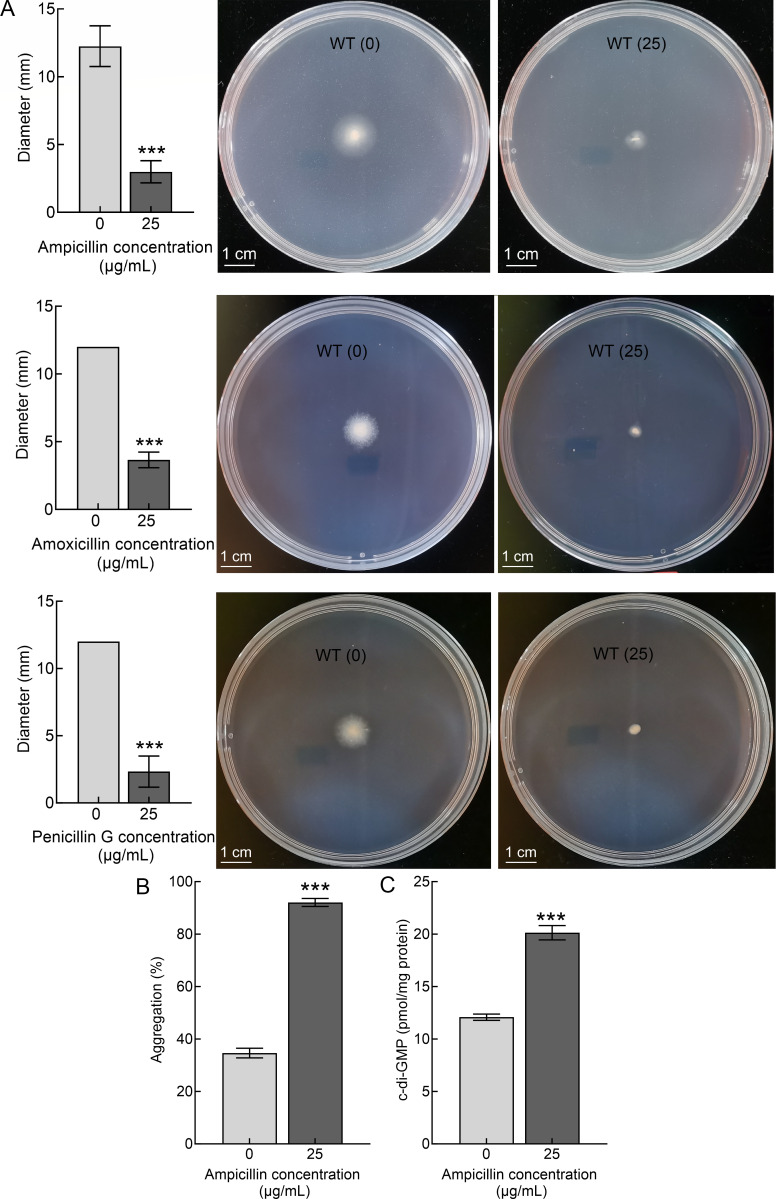
Ampicillin promotes biofilm formation by increasing intracellular c-di-GMP levels. (**A**) The effect of ampicillin, amoxicillin, and penicillin G on the swimming diameter at 48 h. (**B**) The effect of ampicillin on the aggregation at 12 h. (**C**) The effect of ampicillin on the intracellular c-di-GMP level at 24 h. Two-sided Student’s *t*-test was used to analyze the statistical significance (***P < 0.001).

Although all six penicillin antibiotics increase the biofilm formation of *S. putrefaciens* CN32, they have different regulatory effects. Normalized biofilm biomass is increased by low concentrations of cloxacillin, methicillin, and carbenicillin but is repressed by high concentrations. However, an increase in the normalized biofilm biomass of *S. putrefaciens* CN32 was observed at ampicillin, amoxicillin, and penicillin G concentrations ranging from 12.5 to 50 µg/mL. More results showed that *S. putrefaciens* CN32 can grow in a medium containing ampicillin, amoxicillin, or penicillin G at concentrations ranging from 12.5 to 50 µg/mL (Fig. 1E), suggesting that *S. putrefaciens* CN32 is naturally resistant to ampicillin, amoxicillin, and penicillin G. Specifically, although the cell growth prior to the early stationary phase decreased with increasing the three antibiotics concentration, it was similar at all concentrations in the late stationary phase (Fig. 1E). In addition, previous studies have shown that, in some bacteria, swimming motility decreases as biofilm formation increases (41, 42). Thus, swimming motility was determined in the presence and absence of 25 µg/mL of each of the three antibiotics, and the swimming diameter was measured at 48 h. The results showed that the swimming diameter of the bacteria on the plate containing three antibiotics was significantly smaller than that on the plate without antibiotics (Fig. 2A), indicating that the addition of three antibiotics significantly reduces the swimming motility of *S. putrefaciens* CN32. In conclusion, the three antibiotics promote the biofilm formation and repress the swimming motility of *S. putrefaciens* CN32.

### Ampicillin increases intracellular c-di-GMP levels by regulating 16 DGCs/PDEs

Since *S. putrefaciens* CN32 is naturally resistant to ampicillin (Fig. 1E; Fig. S1), and since ampicillin is a frequently used antibiotic in laboratories and has the most significant effect on biofilm formation (Fig. 1A), we used ampicillin to investigate how penicillin antibiotics increase *S. putrefaciens* CN32 biofilm formation. As the addition of 50 µg/mL ampicillin significantly affected the early growth of bacteria (Fig. 1E), a concentration of 25 µg/mL ampicillin was chosen as the subsequent experimental condition. Biofilms have been defined as aggregates of microorganisms in which cells are frequently embedded in a self-produced matrix of extracellular polymeric substances (EPS) that are adherent to each other (aggregation) and/or a surface (1). Thus, the auto-aggregation was determined in the absence and the presence of 25 µg/mL of the ampicillin. The results showed that the aggregation index of *S. putrefaciens* CN32 was significantly increased in the presence of ampicillin (Fig. 2B). Thus, ampicillin increases the biofilm formation and auto-aggregation but represses swimming motility. c-di-GMP is an important second messenger that promotes biofilm formation and auto-aggregation while repressing swimming motility (41, 42). Thus, we investigated whether ampicillin regulated intracellular c-di-GMP levels of *S. putrefaciens* CN32. The result showed that the intracellular c-di-GMP levels were significantly increased after the addition of ampicillin (Fig. 2C). Thus, ampicillin promotes biofilm formation by increasing intracellular c-di-GMP levels in *S. putrefaciens* CN32.

**Fig 3:**
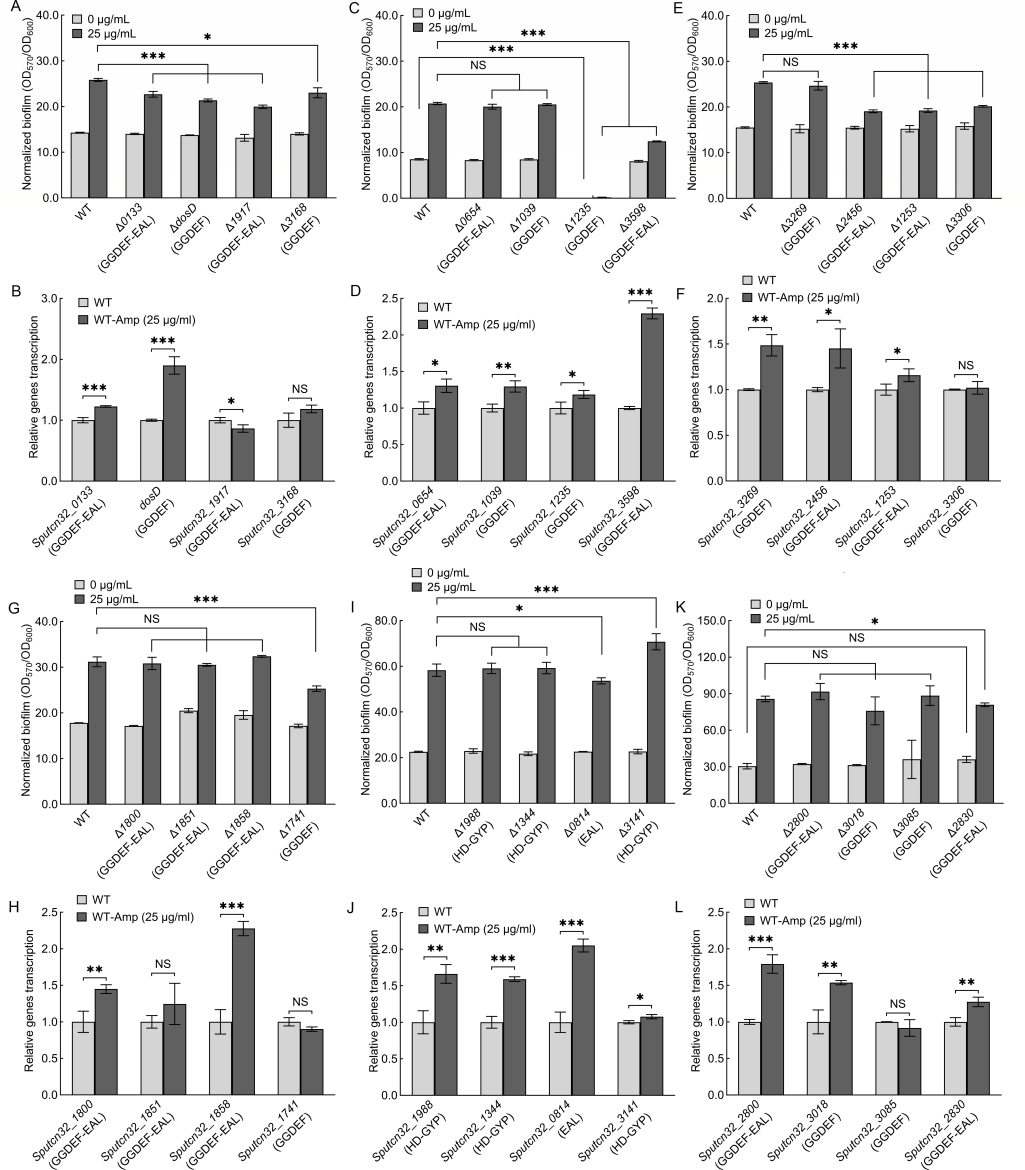
The effect of ampicillin on biofilm formation of DGC/PDE gene deletion mutants. (**A, C, E, G, I, and K**). The effect of ampicillin on biofilm formation at 24 h. (**B, D, F, H, J, and L**). The effect of ampicillin on the transcription of corresponding DGC/PDE genes at 24 h. Two-sided Student’s *t*-test was used to analyze the statistical significance (NS: no significance; *P < 0.05; **P < 0.01; ***P < 0.001).

**Fig 4:**
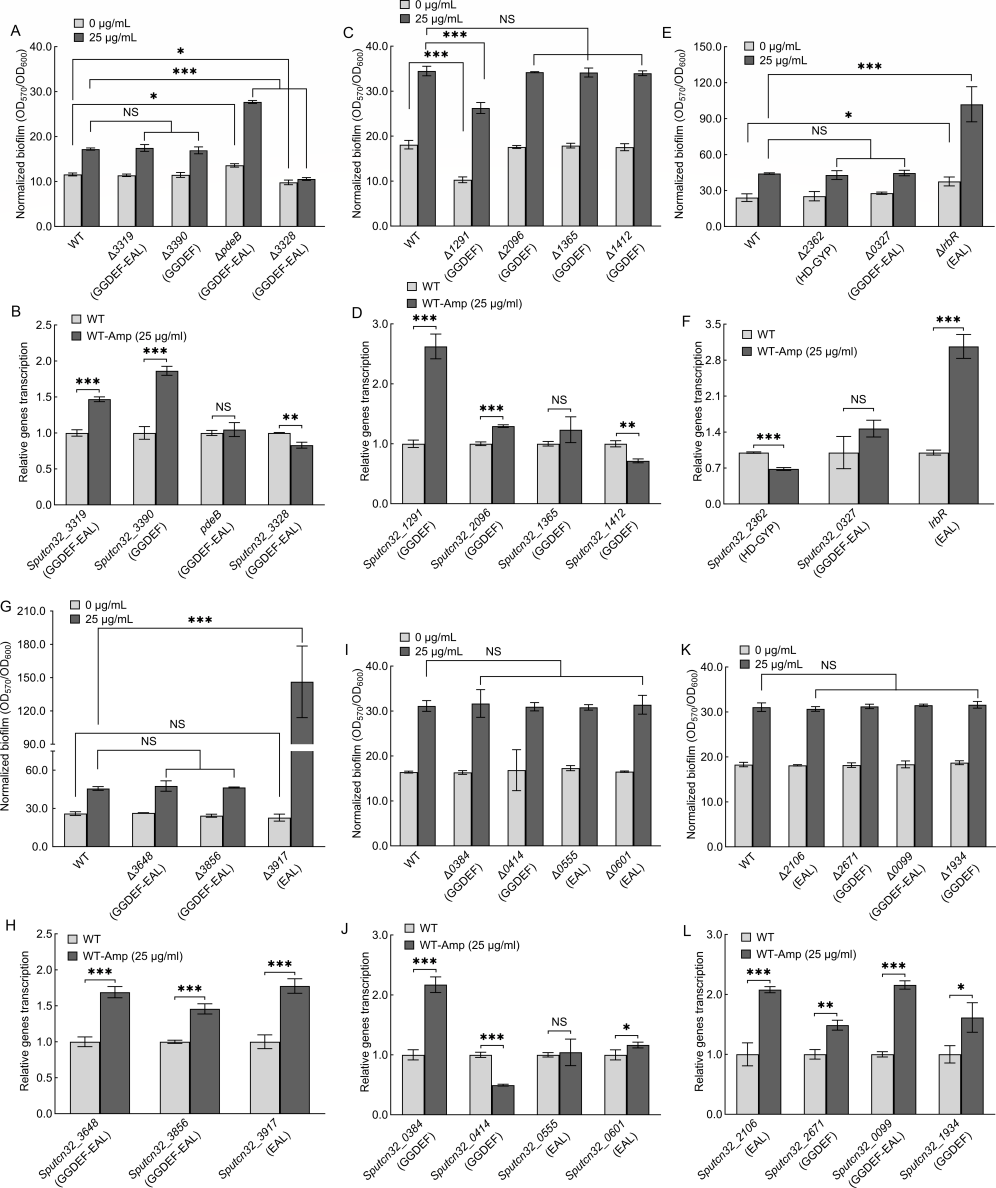
The effect of ampicillin on biofilm formation of DGC/PDE gene deletion mutants. (**A, C, E, G, I, and K**). The effect of ampicillin on biofilm formation at 24 h. (**B, D, F, H, J, and L**). The effect of ampicillin on the transcription of corresponding DGC/PDE genes at 24 h. Two-sided Student’s *t*-test was used to analyze the statistical significance (NS: no significance; *P < 0.05; **P < 0.01; ***P < 0.001).

*S. putrefaciens* CN32 has 47 GGDEF/EAL/HD-GYP domain-containing proteins (Table S1), but only 46 of these genes can be deleted (40). The biofilm formation of all 46 of these mutants was then evaluated in both the absence and presence of 25 µg/mL of ampicillin, and qRT-PCR was performed to determine which DGC/PDE genes were altered in transcription by the addition of ampicillin (Fig. 3 and 4). The 11 DGC/PDE gene deletion mutants include Δ*0133* (Fig. 3A), Δ*dosD* (Fig. 3A), Δ*1917* (Fig. 3A), Δ*3168* (Fig. 3A), Δ*3598* (Fig. 3C), Δ*2456* (Fig. 3E), Δ*1253* (Fig. 3E), Δ*3306* (Fig. 3E), Δ*1741* (Fig. 3G), Δ*0814* (Fig. 3I), and Δ*2830* (Fig. 3K) and exhibited parallel normalized biofilm biomass to WT in the absence of ampicillin, suggesting that these 11 DGCs/PDEs do not regulate biofilm formation in the absence of ampicillin. However, the normalized biofilm biomass of these 11 mutants was lower than that of WT in the presence of ampicillin, suggesting that the deletion of these 11 genes results in ampicillin-increased biofilm biomass failing to reach the same levels in the mutants as in the WT. Of the 11 DGCs/PDEs, DosD contains a GGDEF domain and has been proven to have DGC activity (43). Three proteins (Sputcn32_3168, Sputcn32_3306, and Sputcn32_1741) contain a GGDEF domain, while six proteins (Sputcn32_0133, Sputcn32_1917, Sputcn32_3598, Sputcn32_2456, Sputcn32_1253, and Sputcn32_2830) contain both a GGDEF and an EAL domain (Fig. 3; Table S1). The decreased biofilm biomass of these gene deletion mutants in the presence of ampicillin may be due to these proteins exhibiting DGC activity through their GGDEF domains. Specifically, ampicillin increases the synthesis of c-di-GMP by controlling these enzymes, thereby promoting the biofilm formation. Therefore, once these enzymes are deleted, the biofilm biomass decreases. qRT-PCR results showed that the transcriptional levels of *dosD* (Fig. 3B), *Sputcn32_3598* (Fig. 3D), and *Sputcn32_2456* (Fig. 3F) increased significantly in the presence of ampicillin, suggesting that ampicillin promotes biofilm formation by increasing the transcription of these three DGC genes. The transcriptional levels of the other seven genes were unaffected or minimally affected by the addition of ampicillin (Fig. 3B, D, F, H, J and L), suggesting that ampicillin may regulate these DGCs/PDEs at the translational or post-translational levels rather than the transcriptional level. It is worth mentioning that the addition of ampicillin increases the transcriptional levels of *Sputcn32_0814* (Fig. 3J), and the normalized biofilm biomass of Δ*0814* was slightly lower than that of WT in the presence of ampicillin (Fig. 3I), indicating ampicillin promotes biofilm formation by increasing the transcriptional levels of *Sputcn32_0814*. These results suggest that Sputcn32_0814 should exhibit DGC activity, promoting biofilm formation by raising intracellular c-di-GMP levels, and deleting *Sputcn32_0814* decreases biofilm biomass. However, a contradiction arises here. Sputcn32_0814 only harbors an EAL domain, which should act as a PDE and decrease c-di-GMP levels. This is inconsistent with the above results. It is possible that the regulation of Sputcn32_0814 on biofilm formation is more complex than we thought. In fact, the function of the protein containing the GGDEF or EAL domain is performed not only as a c-di-GMP metabolic enzyme, but also as a c-di-GMP effector (17, 44). For instance, RpfR is a pathogenicity factor of *Burkholderia cenocepacia*, which is not only a PDE but also a c-di-GMP effector (45). At high cell density, the concentration of the quorum sensing signal *Burkholderia* diffusible signal factor (BDSF) increases significantly, which binds to RpfR and triggers its c-di-GMP PDE activity (45). At low cell density, the PDE activity of RfpR cannot be activated due to low concentrations of BDSF (45). When c-di-GMP level is significantly high, RpfR functions as a c-di-GMP effector by binding to c-di-GMP (45). Thus, we hypothesize that the EAL domain may play a role in this regulatory process beyond that of a PDE. In addition, the regulation of DGC/PDE is complex, not only by controlling intracellular c-di-GMP levels (global c-di-GMP signaling) but also by regulating local c-di-GMP signaling (17, 44). In summary, further studies are needed to determine the function of this protein.

The normalized biofilm biomass of Δ*1235*, Δ*3328*, and Δ*1291* was lower than that of WT without the addition of ampicillin (Fig. 3C, 4A and C). Sputcn32_3328 and Sputcn32_1291 have DGC activity *in vivo* (36), and Sputcn32_1235 contains GGDEF domain (Fig. 3C; Table S1). Thus, the three proteins promote biofilm formation by increasing c-di-GMP signaling in the absence of ampicillin, and the biofilm biomass of the three deletion mutants decreases. The normalized biofilm biomass of Δ*1235*, Δ*3328*, and Δ*1291* remained lower than that of WT in the presence of ampicillin, but the situation appears to be different. Δ*1235* does not form a biofilm in either the absence or presence of ampicillin (Fig. 3C). Although ampicillin significantly increased the normalized biofilm biomass of Δ*1291*, the ampicillin-increased biofilm biomass of Δ*1291 was* still lower than that of the ampicillin-increased WT (Fig. 4C). This suggests that ampicillin does not affect the DGC activity of either Sputcn32_1291 or Sputcn32_1235. The ampicillin-increased normalized biofilm biomass of Δ*3328* was significantly lower than that of ampicillin-increased WT (Fig. 4A), suggesting that Sputcn32_3328 has a regulatory role in ampicillin-increased biofilm formation. qRT-PCR results showed that the addition of ampicillin caused slight changes in *Sputcn32_3328* transcription (Fig. 4B), suggesting that ampicillin may regulate *Sputcn32_3328* at the translational or post-translational levels rather than the transcriptional level.

The normalized biofilm biomass of Δ*pdeB* and Δ*lrbR is* higher than that of WT in the absence of ampicillin (Fig. 4A and E), suggesting that PdeB and LrbR negatively regulate biofilm formation. PdeB and LrbR harbor a typical EAL domain and have been shown to degrade c-di-GMP *in vivo* (46, 47). Therefore, deleting *pdeB* or *lrbR* increases intracellular c-di-GMP levels, thereby increasing biofilm biomass. The normalized biofilm biomass of Δ*pdeB* and Δ*lrbR* was still higher than that of WT with the addition of ampicillin (Fig. 4A and E). Most importantly, the normalized biofilm biomass increased by ampicillin was significantly higher for the Δ*pdeB* and Δ*lrbR* mutants than for the WT strain, suggesting that although PdeB and LrbR have the PDE activity in the absence of ampicillin, ampicillin can further enhance their regulatory ability with regard to biofilm formation. The qRT-PCR results showed that the addition of ampicillin did not affect the transcriptional levels of *pdeB* (Fig. 4B) but significantly increased the transcriptional levels of *lrbR* (Fig. 4F), suggesting that ampicillin regulates LrbR at the transcriptional level, but that its regulation of PdeB may occur at the translational or post-translational levels.

Sputcn32_3141 contains a typical HD-GYP domain and Sputcn32_3917 contains a typical EAL domain (Fig. 3I and 4G; Table S1). Thus, both may have PDE activity. However, the normalized biofilm biomass of Δ*3141* and Δ*3917* was similar to that of WT without addition of ampicillin (Fig. 3I and 4G), suggesting that Sputcn32_3141 and Sputcn32_3917 did not have PDE activity in the absence of ampicillin. In addition, the normalized biofilm biomass of Δ*3141* and Δ*3917* was higher than that of WT with the addition of ampicillin (Fig. 3I and 4G), and the normalized biofilm biomass increased by ampicillin was significantly higher for the Δ*3141* and Δ*3917* mutants than for the WT strain (Fig. 3I and 4G). This indicates that the PDE activity of Sputcn32_3141 and Sputcn32_3917 can be induced by the addition of ampicillin, which decreases biofilm formation of WT. The qRT-PCR results showed that the addition of ampicillin did not affect the transcriptional levels of *Sputcn32_3141* but significantly increased the transcriptional levels of *Sputcn32_3917* (Fig. 3J and 4H), suggesting that ampicillin regulates Sputcn32_3917 at the transcriptional level, but that its regulation of Sputcn32_3141 may occur at the translational or post-translational levels.

Although the addition of ampicillin significantly alters the transcriptional levels of most DGC/PDE genes (Fig. 3 and 4), only some of the DGC/PDE deletion mutants exhibit changes in biofilm biomass compared to the WT in the presence of ampicillin (Fig. 3 and 4, and Table 1). This suggests that some DGCs/PDEs exhibit no activity in the presence or absence of ampicillin, resulting in no regulation of biofilm formation, even when their transcriptional levels are altered by ampicillin. Of the 46 DGC/PDEs, 16 c-di-GMP metabolic enzymes are involved in ampicillin-controlled biofilm formation, including DGCs containing the GGDEF domain and PDEs containing the EAL or HD-GYP domain (Table 1). Although the changes in the normalized biofilm biomass of some c-di-GMP metabolic enzyme deletion mutants are minor in the presence of ampicillin, such as Δ*0133*, Δ*2830*, and Δ*0814*, it is the combined action of these enzymes that results in an increase in intracellular c-di-GMP levels.

**TABLE 1:** The c-di-GMP metabolic enzymes regulated by ampicillin

Protein	c-di-GMP metabolic domains	Regulated by ampicillin
Sputcn32_0133	GGDEF-EAL	Potential at the translational or post-translational levels
LrbR	EAL	At transcriptional level
Sputcn32_0814	EAL	At transcriptional level
Sputcn32_1253	GGDEF-EAL	Potential at the translational or post-translational levels
Sputcn32_1741	GGDEF	Potential at the translational or post-translational levels
Sputcn32_1917	GGDEF-EAL	Potential at the translational or post-translational levels
Sputcn32_2456	GGDEF-EAL	At transcriptional level
Sputcn32_2830	GGDEF-EAL	Potential at the translational or post-translational levels
Sputcn32_3141	HD-GYP	Potential at the translational or post-translational levels
Sputcn32_3168	GGDEF	Potential at the translational or post-translational levels
DosD	GGDEF	At transcriptional level
Sputcn32_3306	GGDEF	Potential at the translational or post-translational levels
Sputcn32_3328	GGDEF-EAL	Potential at the translational or post-translational levels
PdeB	GGDEF-EAL	Potential at the translational or post-translational levels
Sputcn32_3598	GGDEF-EAL	At transcriptional level
Sputcn32_3917	EAL	At transcriptional level

### Ampicillin regulates BpfAGD system by increasing intracellular c-di-GMP levels

**Fig 5:**
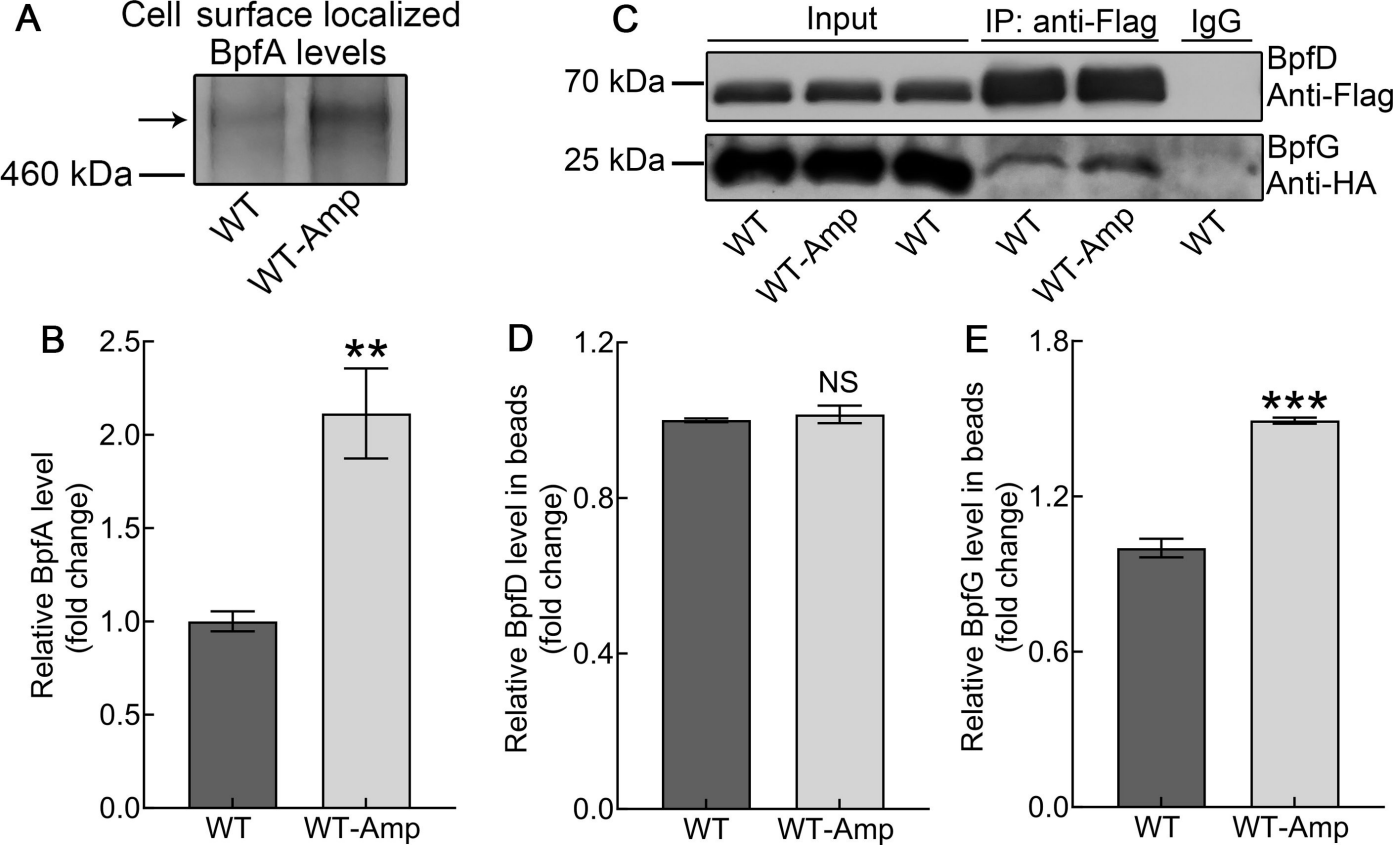
Ampicillin regulates the BpfAGD system of *S. putrefaciens* CN32. (**A**) Western blotting detection of BpfA localization on the cell surface at 24 h. (**B**) Band intensities of panel **A** were quantified using Image J software and normalized to WT. (**C**) Co-IP using antibodies against BpfD, BpfG, and immunoglobulin G at 24 h. IgG, an unimmunized antibody, was used as a negative control to exclude non-specific binding of beads to BpfD. (**D**) Band intensities of BpfD in panel **C** were quantified using Image J software and normalized to WT. (**E**) Band intensities of BpfG in panel **C** were quantified using Image J software and normalized to WT. Two-sided Student’s *t*-test was used to analyze the statistical significance (NS: no significance; **P < 0.01; ***P < 0.001).

In *S. putrefaciens* CN32, intracellular c-di-GMP levels regulate biofilm formation mainly via the BpfAGD system (36, 38, 40). To investigate whether ampicillin regulates the BpfAGD system by controlling intracellular c-di-GMP levels, the levels of BpfA localized on the cell surface and the interaction between BpfD and BpfG were evaluated. The results showed that the levels of BpfA localized on the cell surface were significantly increased (Fig. 5A and B), and the interaction between BpfD and BpfG was stronger in ampicillin-promoted biofilm (Fig. 5C through E), indicating that ampicillin influences the BpfAGD system by controlling intracellular c-di-GMP levels. Thus, ampicillin increases the intracellular c-di-GMP levels to influence the BpfAGD system, thereby inducing biofilm formation.

### Penicillins and carbapenem antibiotics promote biofilm formation of S*. oneidensis* MR-1

**Fig 6:**
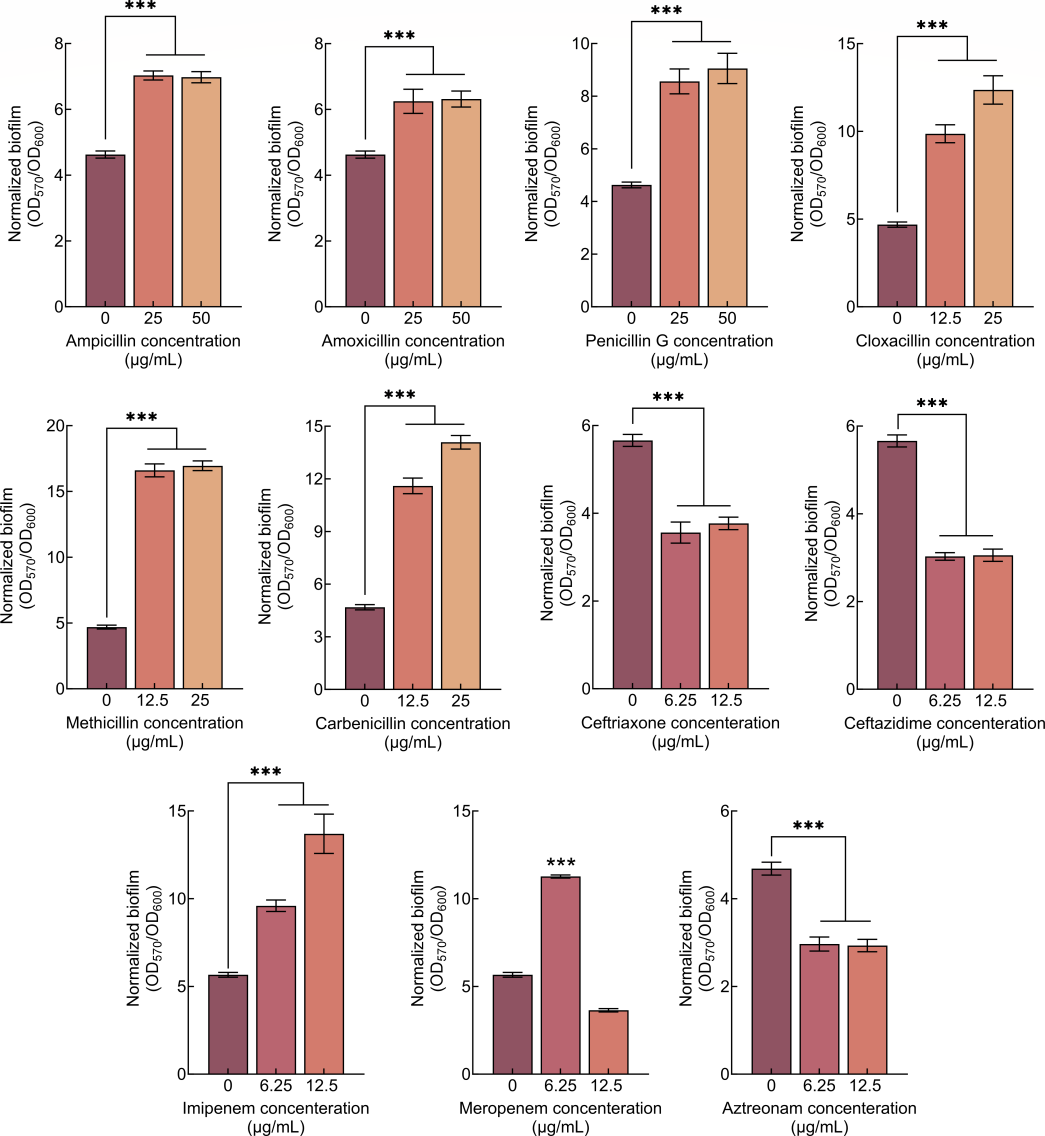
The effect of multiple β-lactam antibiotics on biofilm formation of *S. oneidensis* MR-1 at 24 h. Two-sided Student’s *t*-test was used to analyze the statistical significance (***P < 0.001).

How do β-lactam antibiotics influence the biofilm formation of other *Shewanella* species? In addition to *S. putrefaciens* CN32, *S. oneidensis* MR-1 is one of the most extensively studied model strains. Its c-di-GMP metabolic enzymes and regulatory model of biofilm formation have also been investigated (48, 49). The BpfAGD system is conserved in *S. oneidensi*s MR-1 and plays an important role in regulating biofilm formation (39). The effect of all the above β-lactam antibiotics on biofilm formation by *S. oneidensis* MR-1 was evaluated. The results showed that similar to *S. putrefaciens* CN32, all six penicillin antibiotics induced an increase in normalized biofilm biomass of *S. oneidensis* MR-1 (Fig. 6). Two cephalosporin antibiotics (ceftriaxone and ceftazidime) and one monobactam antibiotic (aztreonam) repress normalized biofilm biomass by *S. oneidensis* MR-1 (Fig. 6). Unlike *S. putrefaciens* CN32, the presence of two carbapenem antibiotics (imipenem and meropenem) increases the normalized biofilm biomass of *S. oneidensis* MR-1 (Fig. 6). Thus, penicillins and carbapenem antibiotics can promote biofilm formation by *S. oneidensis* MR-1.

## DISCUSSION

An increasing number of studies indicate that sub-inhibitory concentrations of antibiotics induce bacterial biofilm formation. For example, sub-inhibitory concentrations of aminoglycoside antibiotics can induce the biofilm formation of *Escherichia coli* and *Pseudomonas aeruginosa* (12). Low concentration β-lactam antibiotics induce the biofilm formation of *Staphylococcus aureus* (50, 51). The sub-inhibitory concentration of aminoglycoside antibiotic gentamicin can induce the biofilm formation of *P. aeruginosa* by increasing the production of extracellular proteins and DNA (52). Although studies have shown that *Shewanella* is highly resistant to β-lactam antibiotics (53–57), there are no studies yet on the impact of β-lactam antibiotics on *Shewanella* biofilm formation.

The present study showed that several penicillin antibiotics increase the biofilm formation of *S. putrefaciens* CN32, whereas the other three classes of β-lactam antibiotic do not have this effect. However, the antibiotics that we used are only some commonly used types of β-lactam antibiotic, which cannot represent all of them. As ampicillin is commonly used in laboratories and can induce an increase in biofilm formation at multiple concentrations, it was chosen as the antibiotic to study the mechanism by which penicillin antibiotics increases biofilm formation. Ampicillin increases intracellular c-di-GMP levels by regulating 16 DGCs/PDEs, which in turn regulate the BpfAGD system and control biofilm formation. *S. putrefaciens* CN32 contains 47 DGCs/PDEs, 16 of which are involved in ampicillin-regulated biofilm formation. While most of the 16 DGCs/PDEs have signal-sensing domains at their N-terminus, it is improbable that a single antibiotic could activate them all. Previous studies showed that antibiotics can regulate many bacterial physiological processes, thereby inducing biofilm formation (13, 14). Thus, we speculate that some DGCs/PDEs respond directly to ampicillin, while the response of others may be mediated by altered physiological processes.

Future research should address the following questions. (i) Similar to ampicillin, amoxicillin and penicillin G increase biofilm formation of *S. putrefaciens* CN32 at various concentrations. Further research is needed to establish whether the regulatory mechanism by which amoxicillin and penicillin G increase biofilm formation is similar to that of ampicillin. (ii) The other three penicillin antibiotics induce biofilm formation of *S. putrefaciens* CN32 at low concentrations but repress it at high concentrations. It is necessary to determine whether the regulatory mechanism by which the three antibiotics increase biofilm formation at low concentrations is similar to that of ampicillin. (iii) Previous studies showed that antibiotics can regulate many bacterial physiological processes, thereby inducing biofilm formation (13, 14). Future studies will investigate which DGC/PDEs respond directly to ampicillin and which are associated with other physiological processes. This will link c-di-GMP metabolic enzymes to ampicillin and other physiological functions. (iv) The BpfAGD system also plays an important role in the biofilm formation of *S. oneidensis* MR-1 and some other *Shewanella* (35, 37). Further studies are needed to establish whether ampicillin increases *S. oneidensis* MR-1 biofilm formation in a manner similar to that identified in *S. putrefaciens* CN32. (v) Unlike *S. putrefaciens* CN32, two carbapenem antibiotics increase the biofilm formation of *S. oneidensis* MR-1. *S. oneidensis* MR-1 has 98 predicted c-di-GMP metabolic enzymes (58), which is far more than in *S. putrefaciens* CN32. Further research is required to determine whether the increased number of DGCs/PDEs is responsible for the increased biofilm formation observed in response to carbapenem antibiotics.

It is of great significance to understand which antibiotics different bacteria are resistant to and which can induce them to form biofilms. (i) Guidance on the correct use of antibiotics. Once a specific bacterial infection has been identified, it is important to avoid using antibiotics that cause bacterial resistance and induce biofilm formation, both in medical treatment and in agricultural production. (ii) A better understanding of the molecular mechanisms underlying bacterial resistance and antibiotic-induced biofilm formation could lay the theoretical groundwork for controlling biofilms in the future.

## MATERIALS AND METHODS

### Bacterial strains and growth conditions

*S. putrefaciens* CN32 and its derivatives used in this study are listed in Table S2, which were grown in LB medium (consisting of 1% tryptone, 1% NaCl, and 0.5% yeast extract) and MM1 medium (30 mM HEPES, 1.34 mM KCl, 28.04 mM NH_4_Cl, 4.35 mM NaH_2_PO_4_, 7.5 mM NaOH, adjusted to pH 7.0, supplemented with 20 mM sodium lactate, 0.68 mM CaCl_2_, and trace amounts of amino acids, minerals, and vitamins [47]). The growth conditions were described previously (40). When necessary, gradient concentrations of different antibiotics were added into the medium.

### Biofilm microtiter plate assay

The LB-cultured *S. putrefaciens* CN32 and its derivatives seeds were diluted in MM1 medium to OD_600_ ~0.01, 100 µL was aliquoted into 96-well cell culture plates (NEST, China). Biofilm formation was assessed using different concentrations of different antibiotics at 24 h. The static biofilm formation assay (OD570) was performed using the 96-well plate method described elsewhere (36). The OD_600_ of normalized biofilm biomass (the ratio of biofilm biomass to cell growth, OD_570_/OD_600_) was also detected in 96-well cell culture plates. The bacteria were cultured in MM1 medium without 0.68 mM CaCl_2_. The antibiotics information is listed in Table S3.

### Determination of aggregation index

The LB-cultured *S. putrefaciens* CN32 seeds were transferred to MM1 medium at an inoculum of 1% and incubated at 30°C at 200 rpm for 12 h. When necessary, ampicillin was added into the medium. The aggregation index of *S. putrefaciens* CN32 was measured according to an established method (59). Briefly, 10 mL of an *S. putrefaciens* CN32 culture in MM1 medium was rigorously vortexed to destroy aggregates, and the OD_600_ of this suspension was determined. This was defined as OD_total_. Another 10 mL of the culture was centrifuged, without vortex treatment, for 2 min at 650 × *g*, and the OD_600_ was determined. This was defined as OD_supernatant_. The aggregation was (OD_total_ − OD_supernatant_)/OD_total_ (59).

### c-di-GMP measurement

The c-di-GMP concentration of *S. putrefaciens* CN32 was measured according to an established method (40). Briefly, the cells were harvested at 24 h, followed by lysis using B-PER bacterial protein extraction reagent (ThermoFisher Scientific, USA). After centrifugation, the supernatant of the lysis solution was sampled for the determination of c-di-GMP concentration using a c-di-GMP ELISA kit (Cayman Chemical, USA) (36, 40), while the total protein concentration was determined using a Quick Start Bradford 1 × dye reagent (BioRad, USA).

### RNA extraction and real-time RT-PCR (qRT-PCR) assay

The TRIzol method was used to extract RNA from cells cultured in 96-well plates at the appropriate times. In accordance with the manufacturer’s protocol (Promega, USA), 2 µg of total RNA was reverse transcribed into cDNA, which was subsequently utilized as a template for the qRT-PCR assay. The qRT-PCR assay was performed using SYBR Green qPCR Mix (Biosharp, China) based on an established method (36). The primers used in qRT-PCR analysis were listed in Table S4.

### Co-immunoprecipitation (Co-IP) assay

Strains grown in 96-well plates at 24 h were harvested, washed once with PBS. Co-IP was performed to verify the interaction between Flag-tagged BpfD and HA-tagged BpfG and 5 µM c-di-GMP was added, which was performed using an established method (36). The bound protein complex was eluted from beads using sample loading buffer and analyzed by western blotting.

### BpfA localization assay

Strains grown in 96-well plates at 24 h were harvested. The collection of BpfA on cell surfaces was determined using an established method (36). The BpfA-containing supernatant fraction was determined by western blotting.

### Western blotting

Total protein concentration was measured using a Quick Start Bradford 1× dye reagent (Bio-Rad, USA). Total proteins from different strains were adjusted to equal amounts for western blotting assay, which was performed using an established method (36). BpfA on cell surface was separated by 5% SDS-PAGE gels and HiMark pre-stained protein standard (ThermoFisher Scientific, USA) was used to identify the BpfA molecular weight. The BpfD and BpfG in the Co-IP assay, which were eluted from beads, were separated by 12% SDS-PAGE gels, and PageRuler pre-stained protein ladder (ThermoFisher Scientific, USA) was used to identify the protein molecular weight. The target protein was then detected using the eECL Western Blotting Kit (CoWin Biosciences, China). Primary antibody: Monoclonal anti-Flag M2 antibody produced in mouse (SigmaAldrich, USA); anti-Flag antibody [DYKDDDDK-Tag (3B9) mAb] (Abmart, China); Anti HA-tag mouse monoclonal antibody (CoWin Biosciences, China). HRP-conjugated secondary antibody: Goat anti-mouse IgG HRP conjugated secondary antibody (CoWin Biosciences, China); Goat Anti-Mouse IgG-Fc HRP conjugated secondary antibody (SinoBiological, China)

### Swimming assay

The swimming motility was measured according to an established method (60, 61). A single colony incubated on an LB plate at 30°C for 24 h was picked with a needle syringe, stabbed into the center of soft agar containing MM1 medium and 0.3% agarose, and then incubated at 30°C. Ampicillin was added to the medium when necessary. Swimming diameters were measured at 48 hours after incubation.

### Statistics and reproducibility

Statistical analyses were performed using GraphPad Prism 10. Three independent samples were used for each trial and the data are presented as a mean ± SD. Statistical significance was determined using two-sided Student’s *t* test. *P*-values are reported using the following symbolic representation: NS (no significance) *P* > 0.05, *P < 0.05; **P < 0.01; ***P < 0.001.

## SUPPLEMENTAL MATERIAL

Supplemental material spectrum.02906-25-s0001.docx Fig. S1; Tables S1 to S4.

ASM does not own the copyrights to Supplemental Material that may be linked to, or accessed through, an article. The authors have granted ASM a non-exclusive, world-wide license to publish the Supplemental Material files. Please contact the corresponding author directly for reuse.

## References

[1] Flemming HC, Wingender J, Szewzyk U, Steinberg P, Rice SA, Kjelleberg S. 2016. Biofilms: an emergent form of bacterial life. Nat Rev Microbiol 14:563–575. doi:10.1038/nrmicro.2016.94 27510863

[2] Ma LZ, Wang D, Liu Y, Zhang Z, Wozniak DJ. 2022. Regulation of biofilm exopolysaccharide biosynthesis and degradation in Pseudomonas aeruginosa. Annu Rev Microbiol 76:413–433. doi:10.1146/annurev-micro-041320-111355 35655342

[3] Flemming H-C, van Hullebusch ED, Neu TR, Nielsen PH, Seviour T, Stoodley P, Wingender J, Wuertz S. 2023. The biofilm matrix: multitasking in a shared space. Nat Rev Microbiol 21:70–86. doi:10.1038/s41579-022-00791-0 36127518

[4] Karygianni L, Ren Z, Koo H, Thurnheer T. 2020. Biofilm matrixome: extracellular components in structured microbial communities. Trends Microbiol 28:668–681. doi:10.1016/j.tim.2020.03.016 32663461

[5] Suarez C, Rosenqvist T, Dimitrova I, Sedlacek CJ, Modin O, Paul CJ, Hermansson M, Persson F. 2024. Biofilm colonization and succession in a full-scale partial nitritation-anammox moving bed biofilm reactor. Microbiome 12:51. doi:10.1186/s40168-024-01762-8 38475926 PMC10935808

[6] Ciofu O, Moser C, Jensen PØ, Høiby N. 2022. Tolerance and resistance of microbial biofilms. Nat Rev Microbiol 20:621–635. doi:10.1038/s41579-022-00682-4 35115704

[7] Yan J, Bassler BL. 2019. Surviving as a community: antibiotic tolerance and persistence in bacterial biofilms. Cell Host Microbe 26:15–21. doi:10.1016/j.chom.2019.06.002 31295420 PMC6629468

[8] Davies D. 2003. Understanding biofilm resistance to antibacterial agents. Nat Rev Drug Discov 2:114–122. doi:10.1038/nrd1008 12563302

[9] Cordisco E, Serra DO. 2025. Moonlighting antibiotics: the extra job of modulating biofilm formation. Trends Microbiol 33:459–471. doi:10.1016/j.tim.2024.12.011 39828459

[10] Andersson DI, Hughes D. 2014. Microbiological effects of sublethal levels of antibiotics. Nat Rev Microbiol 12:465–478. doi:10.1038/nrmicro3270 24861036

[11] Bleich R, Watrous JD, Dorrestein PC, Bowers AA, Shank EA. 2015. Thiopeptide antibiotics stimulate biofilm formation in Bacillus subtilis. Proc Natl Acad Sci USA 112:3086–3091. doi:10.1073/pnas.1414272112 25713360 PMC4364203

[12] Hoffman LR, D’Argenio DA, MacCoss MJ, Zhang Z, Jones RA, Miller SI. 2005. Aminoglycoside antibiotics induce bacterial biofilm formation. Nature 436:1171–1175. doi:10.1038/nature03912 16121184

[13] Yang S, Li D, Fu S, Zheng J, Zhu Y, Li H, Zeng H, Zhang J. 2025. Decoding the effect of antibiotics on biofilm formation in biofilters. J Environ Manage 385:125698. doi:10.1016/j.jenvman.2025.125698 40347873

[14] Wu S, Li X, Gunawardana M, Maguire K, Guerrero-Given D, Schaudinn C, Wang C, Baum MM, Webster P. 2014. Beta-lactam antibiotics stimulate biofilm formation in non-typeable Haemophilus influenzae by up-regulating carbohydrate metabolism. PLoS One 9:e99204. doi:10.1371/journal.pone.0099204 25007395 PMC4090067

[15] Sauer K, Stoodley P, Goeres DM, Hall-Stoodley L, Burmølle M, Stewart PS, Bjarnsholt T. 2022. The biofilm life cycle: expanding the conceptual model of biofilm formation. Nat Rev Microbiol 20:608–620. doi:10.1038/s41579-022-00767-0 35922483 PMC9841534

[16] Jenal U, Reinders A, Lori C. 2017. Cyclic di-GMP: second messenger extraordinaire. Nat Rev Microbiol 15:271–284. doi:10.1038/nrmicro.2016.190 28163311

[17] Dahlstrom KM, O’Toole GA. 2017. A symphony of cyclases: specificity in diguanylate cyclase signaling. Annu Rev Microbiol 71:179–195. doi:10.1146/annurev-micro-090816-093325 28645224 PMC5936083

[18] Plate L, Marletta MA. 2012. Nitric oxide modulates bacterial biofilm formation through a multicomponent cyclic-di-GMP signaling network. Mol Cell 46:449–460. doi:10.1016/j.molcel.2012.03.023 22542454 PMC3361614

[19] Sarenko O, Klauck G, Wilke FM, Pfiffer V, Richter AM, Herbst S, Kaever V, Hengge R, Shuman HA, Jenal U, Wolfe A. 2017. More than enzymes that make or break cyclic di-GMP--local signaling in the interactome of GGDEF/EAL domain proteins of Escherichia coli. mBio 8:e01639-17. doi:10.1128/mBio.01639-17 29018125 PMC5635695

[20] Arad E, Pedersen KB, Malka O, Mambram Kunnath S, Golan N, Aibinder P, Schiøtt B, Rapaport H, Landau M, Jelinek R. 2023. Staphylococcus aureus functional amyloids catalyze degradation of β-lactam antibiotics. Nat Commun 14:8198. doi:10.1038/s41467-023-43624-1 38081813 PMC10713593

[21] Lewis K, Lee RE, Brötz-Oesterhelt H, Hiller S, Rodnina MV, Schneider T, Weingarth M, Wohlgemuth I. 2024. Sophisticated natural products as antibiotics. Nature 632:39–49. doi:10.1038/s41586-024-07530-w 39085542 PMC11573432

[22] Lima LM, Silva BNM da, Barbosa G, Barreiro EJ. 2020. β-lactam antibiotics: an overview from a medicinal chemistry perspective. Eur J Med Chem 208:112829. doi:10.1016/j.ejmech.2020.112829 33002736

[23] Liu J, Yang L, Hou Y, Soteyome T, Zeng B, Su J, Li L, Li B, Chen D, Li Y, Wu A, Shirtliff ME, Harro JM, Xu Z, Peters BM. 2018. Transcriptomics study on Staphylococcus aureus biofilm under low concentration of ampicillin. Front Microbiol 9:2413. doi:10.3389/fmicb.2018.02413 30425687 PMC6218852

[24] Lemaire ON, Méjean V, Iobbi-Nivol C. 2020. The Shewanella genus: ubiquitous organisms sustaining and preserving aquatic ecosystems. FEMS Microbiol Rev 44:155–170. doi:10.1093/femsre/fuz031 31922549

[25] Hau HH, Gralnick JA. 2007. Ecology and biotechnology of the genus Shewanella. Annu Rev Microbiol 61:237–258. doi:10.1146/annurev.micro.61.080706.093257 18035608

[26] Tiedje JM. 2002. Shewanella—the environmentally versatile genome. Nat Biotechnol 20:1093–1094. doi:10.1038/nbt1102-1093 12410251

[27] Yoon S, Cruz-García C, Sanford R, Ritalahti KM, Löffler FE. 2015. Denitrification versus respiratory ammonification: environmental controls of two competing dissimilatory NO_3_^−^/NO_2_^−^ reduction pathways in Shewanella loihica strain PV-4. ISME J 9:1093–1104. doi:10.1038/ismej.2014.201 25350157 PMC4409154

[28] Vogel BF, Jørgensen K, Christensen H, Olsen JE, Gram L. 1997. Differentiation of Shewanella putrefaciens and Shewanella alga on the basis of whole-cell protein profiles, ribotyping, phenotypic characterization, and 16S rRNA gene sequence analysis. Appl Environ Microbiol 63:2189–2199. doi:10.1128/aem.63.6.2189-2199.1997 9172338 PMC168511

[29] Jiang X, Wang X, Li L, Niu C, Pei C, Zhu L, Kong X. 2022. Identification of Shewanella putrefaciens as a novel pathogen of the largemouth bass (Micropterus salmoides) and histopathological analysis of diseased fish. Front Cell Infect Microbiol 12:1042977. doi:10.3389/fcimb.2022.1042977 36325466 PMC9618692

[30] Yi Z, Yan J, Ding Z, Xie J. 2022. The HD-GYP domain protein of Shewanella putrefaciens YZ08 regulates biofilm formation and spoilage activities. Food Res Int 157:111466. doi:10.1016/j.foodres.2022.111466 35761698

[31] Li P, Mei J, Tan M, Xie J. 2022. Effect of CO2 on the spoilage potential of Shewanella putrefaciens target to flavour compounds. Food Chem 397:133748. doi:10.1016/j.foodchem.2022.133748 35905618

[32] Muller S, Bonin S, Schneider R, Kruger M, Quick S, Schrottner P. 2022. Shewanella putrefaciens, a rare human pathogen: a review from a clinical perspective. Front Cell Infect Microbiol 12:1033639. 36817694 10.3389/fcimb.2022.1033639 PMC9933709

[33] Sher S, Richards GP, Parveen S, Williams HN. 2025. Characterization of antibiotic resistance in Shewanella species: an emerging pathogen in clinical and environmental settings. Microorganisms 13:1115. doi:10.3390/microorganisms13051115 40431288 PMC12114352

[34] Lloyd NA, Nazaret S, Barkay T. 2018. Whole genome sequences to assess the link between antibiotic and metal resistance in three coastal marine bacteria isolated from the mummichog gastrointestinal tract. Mar Pollut Bull 135:514–520. doi:10.1016/j.marpolbul.2018.07.051 30301067

[35] Zhou G, Yuan J, Gao H. 2015. Regulation of biofilm formation by BpfA, BpfD, and BpfG in Shewanella oneidensis. Front Microbiol 6:790. doi:10.3389/fmicb.2015.00790 26300859 PMC4523816

[36] Liu C, Sun D, Liu J, Chen Y, Zhou X, Ru Y, Zhu J, Liu W. 2022. cAMP and c-di-GMP synergistically support biofilm maintenance through the direct interaction of their effectors. Nat Commun 13:1493. doi:10.1038/s41467-022-29240-5 35315431 PMC8938473

[37] Collins AJ, Smith TJ, Sondermann H, O’Toole GA. 2020. From input to output: the Lap/c-di-GMP biofilm regulatory circuit. Annu Rev Microbiol 74:607–631. doi:10.1146/annurev-micro-011520-094214 32689917 PMC8966053

[38] Liu C, Shi R, Jensen MS, Zhu J, Liu J, Liu X, Sun D, Liu W. 2024. The global regulation of c-di-GMP and cAMP in bacteria. mLife 3:42–56. doi:10.1002/mlf2.12104 38827514 PMC11139211

[39] Theunissen S, De Smet L, Dansercoer A, Motte B, Coenye T, Van Beeumen JJ, Devreese B, Savvides SN, Vergauwen B. 2010. The 285 kDa Bap/RTX hybrid cell surface protein (SO4317) of Shewanella oneidensis MR-1 is a key mediator of biofilm formation. Res Microbiol 161:144–152. doi:10.1016/j.resmic.2009.12.002 20034561

[40] Sun D, Liu X, Zhang Y, Shi R, Ru Y, Zhou X, Chen Y, Yang J, Liu J, Zhu J, Liu C, Liu W. 2025. Local c-di-GMP signaling, triggered by cross-regulation of cAMP-CRP and c-di-GMP, controls biofilm formation under nutrient limitation. Proc Natl Acad Sci USA 122: e2516964122. doi:10.1073/pnas.2516964122 PMC12415220 40854124

[41] Chen X, Wang L, Sourjik V. 2025. Swimming or sessile: the interplay between c-di-GMP signalling and flagellar motility. Curr Opin Microbiol 87:102632. doi:10.1016/j.mib.2025.102632 40638951

[42] Khan F, Jeong GJ, Tabassum N, Kim YM. 2023. Functional diversity of c-di-GMP receptors in prokaryotic and eukaryotic systems. Cell Commun Signal 21:259. doi:10.1186/s12964-023-01263-5 37749602 PMC10519070

[43] Cheng YY, Wu C, Wu JY, Jia HL, Wang MY, Wang HY, Zou SM, Sun RR, Jia R, Xiao YZ, Schottel JL. 2017. FlrA represses transcription of the biofilm-associated bpfA operon in Shewanella putrefaciens. Appl Environ Microbiol 83:e02410-16. doi:10.1128/AEM.02410-16 27986717 PMC5288817

[44] Hengge R. 2021. High-specificity local and global c-di-GMP signaling. Trends Microbiol 29:993–1003. doi:10.1016/j.tim.2021.02.003 33640237

[45] Yang C, Cui C, Ye Q, Kan J, Fu S, Song S, Huang Y, He F, Zhang L-H, Jia Y, Gao YG, Harwood CS, Deng Y. 2017. Burkholderia cenocepacia integrates cis-2-dodecenoic acid and cyclic dimeric guanosine monophosphate signals to control virulence. Proc Natl Acad Sci USA 114:13006–13011. doi:10.1073/pnas.1709048114 29158389 PMC5724260

[46] Rossmann FM, Rick T, Mrusek D, Sprankel L, Dörrich AK, Leonhard T, Bubendorfer S, Kaever V, Bange G, Thormann KM. 2019. The GGDEF domain of the phosphodiesterase PdeB in Shewanella putrefaciens mediates recruitment by the polar landmark protein HubP. J Bacteriol 201:e00534-18. doi:10.1128/JB.00534-18 30670544 PMC6416913

[47] Liu C, Yang J, Liu L, Li B, Yuan H, Liu W. 2017. Sodium lactate negatively regulates Shewanella putrefaciens CN32 biofilm formation via a three component regulatory system (LrbS-LrbALrbR). Appl Environ Microbiol 83:e00712-17. doi:10.1128/AEM.00712-17 28500045 PMC5494631

[48] Rakshe S, Leff M, Spormann AM. 2011. Indirect modulation of the intracellular c-Di-GMP level in Shewanella oneidensis MR-1 by MxdA. Appl Environ Microbiol 77:2196–2198. doi:10.1128/AEM.01985-10 21278272 PMC3067315

[49] Chao L, Rakshe S, Leff M, Spormann AM. 2013. PdeB, a cyclic Di-GMP-specific phosphodiesterase that regulates Shewanella oneidensis MR-1 motility and biofilm formation. J Bacteriol 195:3827–3833. doi:10.1128/JB.00498-13 23794617 PMC3754596

[50] Azzam A, Shawky RM, El-Mahdy TS. 2024. Sub-inhibitory concentrations of ceftriaxone induce morphological alterations and PIA-independent biofilm formation in Staphylococcus aureus. Braz J Microbiol 55:297–308. doi:10.1007/s42770-023-01177-x 37979131 PMC10920565

[51] Kaplan JB, Izano EA, Gopal P, Karwacki MT, Kim S, Bose JL, Bayles KW, Horswill AR. 2012. Low levels of β-lactam antibiotics induce extracellular DNA release and biofilm formation in Staphylococcus aureus. mBio 3:e00198-12. doi:10.1128/mBio.00198-12 22851659 PMC3419523

[52] Kumar A, Saha SK, Banerjee P, Prasad K, Sengupta TK. 2023. Antibiotic-induced biofilm formations in Pseudomonas aeruginosa strains KPW.1-S1 and HRW.1-S3 are associated with increased production of eDNA and exoproteins, increased ROS generation, and increased cell surface hydrophobicity. Curr Microbiol 81:11. doi:10.1007/s00284-023-03495-7 37978089

[53] Johnson TN, Richards GP, Parveen S. 2025. Prevalence, antibiotic resistance, and control of pathogenic Shewanella in seafoods. J Food Prot 88:100570. doi:10.1016/j.jfp.2025.100570 40582618

[54] Murhekar S, Wright MH, Greene AC, Brownlie JC, Cock IE. 2017. Inhibition of Shewanella spp. growth by Syzygium australe and Syzygium luehmannii extracts: natural methods for the prevention of fish spoilage. J Food Sci Technol 54:3314–3326. doi:10.1007/s13197-017-2782-6 28974817 PMC5602996

[55] Gao H, Obraztova A, Stewart N, Popa R, Fredrickson JK, Tiedje JM, Nealson KH, Zhou J. 2006. Shewanella loihica sp. nov., isolated from iron-rich microbial mats in the Pacific Ocean. Int J Syst Evol Microbiol 56:1911–1916. doi:10.1099/ijs.0.64354-0 16902030

[56] Yin J, Sun L, Dong Y, Chi X, Zhu W, Qi S, Gao H. 2013. Expression of blaA underlies unexpected ampicillin-induced cell lysis of Shewanella oneidensis. PLoS One 8:e60460. doi:10.1371/journal.pone.0060460 23555975 PMC3610667

[57] Yin J, Sun Y, Mao Y, Jin M, Gao H. 2015. PBP1a/LpoA but not PBP1b/LpoB are involved in regulation of the major β-lactamase gene blaA in Shewanella oneidensis. Antimicrob Agents Chemother 59:3357–3364. doi:10.1128/AAC.04669-14 25824223 PMC4432149

[58] Fredrickson JK, Romine MF, Beliaev AS, Auchtung JM, Driscoll ME, Gardner TS, Nealson KH, Osterman AL, Pinchuk G, Reed JL, Rodionov DA, Rodrigues JLM, Saffarini DA, Serres MH, Spormann AM, Zhulin IB, Tiedje JM. 2008. Towards environmental systems biology of Shewanella. Nat Rev Microbiol 6:592–603. doi:10.1038/nrmicro1947 18604222

[59] De Windt W, Gao H, Krömer W, Van Damme P, Dick J, Mast J, Boon N, Zhou J, Verstraete W. 2006. AggA is required for aggregation and increased biofilm formation of a hyper-aggregating mutant of Shewanella oneidensis MR-1. Microbiology (Reading, Engl) 152:721–729. doi:10.1099/mic.0.28204-0 16514152

[60] Natarajan O, Gibboney SL, Young MN, Lim SJ, Nguyen F, Pluta N, Atkinson CGF, Liberti A, Kees ED, Leigh BA, Breitbart M, Gralnick JA, Dishaw LJ. 2025. Prophage regulation of Shewanella fidelis 3313 motility and biofilm formation with implications for gut colonization dynamics in Ciona robusta. eLife 14:RP103107. doi:10.7554/eLife.103107 40956701 PMC12440353

[61] Kearns DB. 2010. A field guide to bacterial swarming motility. Nat Rev Microbiol 8:634–644. doi:10.1038/nrmicro2405 20694026 PMC3135019

