# Supplementary material for: Ampicillin promotes the biofilm formation of Shewanella putrefaciens through the c-di-GMP-regulated BpfAGD system
Source: Microbiol Spectr. 2025 Nov 19;14(1):e02906-25. doi: 10.1128/spectrum.02906-25 (PMC12772357; doi:10.1128/spectrum.02906-25)
Supplement: Supplemental material — Fig. S1; Tables S1 to S4. [file spectrum.02906-25-s0001.docx]

**Supplementary information**

**Ampicillin promotes the biofilm formation of *Shewanella putrefaciens* through the c-di-GMP regulated BpfAGD system**

Rui Shi^a,^ *, Di Sun^a,^ *, Jiawen Liu^a^, Jing Yang^a^, Jingrong Zhu^a^, Cong Liu^a,^ **^#^**, Weijie Liu^a,^ **^#^**

^a^ Department of Microbiology, School of Life Sciences, Jiangsu Normal University, Xuzhou 221116, Jiangsu Province, China

***** These authors contributed equally to this article. The order of authors was determined by the alphabetical order of their family names.

**^#^ Corresponding:** Cong Liu, Tel: +86 18610712643, E-mail: [liucong0426@126.com](mailto:liucong0426@126.com); Weijie Liu, Tel: +86 18252161264, E-mail: [leonliu2013@126.com](mailto:leonliu2013@126.com%20(WJ%20Liu))


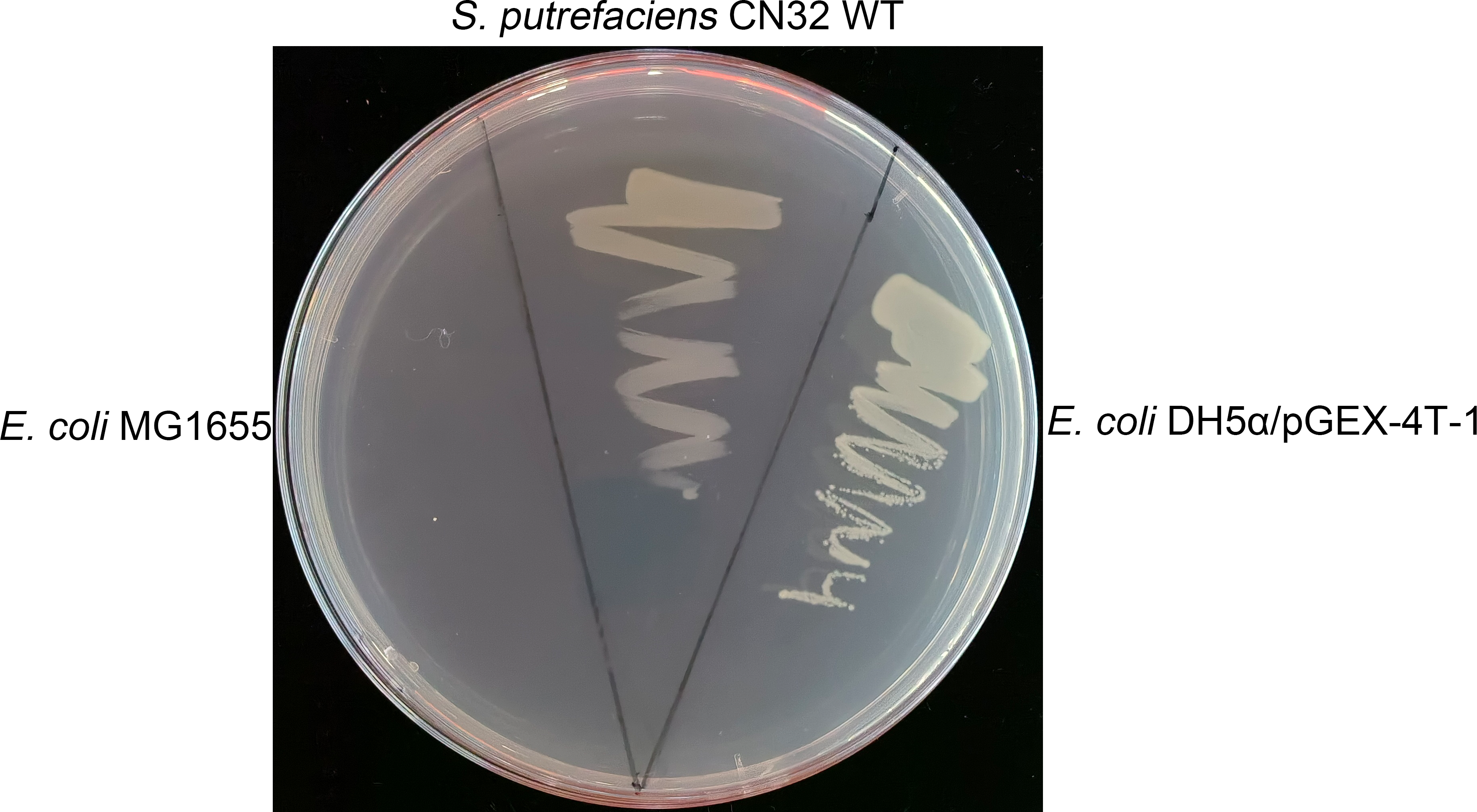


**Fig. S1 *S. putrefaciens* CN32 is naturally resistant to ampicillin.** On the left of the plate is the negative control, which is an ampicillin-sensitive strain of *E. coli* (MG1655). On the right is the positive control, which is an ampicillin-resistant strain of *E. coli* (DH5α/pGEX-4T-1) containing plasmids with an ampicillin resistance gene. In the middle of the plate is *S. putrefaciens* CN32. The medium on the plate is LB supplemented with 100 µg/mL ampicillin.

**Table S1. 47 DGCs/PDEs of *S. putrefaciens* CN32**

| Protein | GGDEF/EAL/HD-GYP | Other domains |
| --- | --- | --- |
| Sputcn32_0099 | GGDEF-EAL | PAS |
| Sputcn32_0133 | GGDEF-EAL | HAMP-PAS-PAC |
| LrbR | EAL | REC |
| Sputcn32_0327 | GGDEF-EAL | PAS |
| Sputcn32_0384 | GGDEF |  |
| Sputcn32_0414 | GGDEF |  |
| Sputcn32_0498 (failed deleted) | GGDEF |  |
| Sputcn32_0555 | EAL |  |
| Sputcn32_0601 | EAL |  |
| Sputcn32_0654 | GGDEF-EAL | GAF |
| Sputcn32_0814 | EAL |  |
| Sputcn32_1039 | GGDEF | GAF |
| Sputcn32_1235 | GGDEF | TPR |
| Sputcn32_1253 | GGDEF-EAL | PAS-PAC |
| Sputcn32_1291 | GGDEF |  |
| Sputcn32_1344 | HD-GYP | GAF-CBS |
| Sputcn32_1365 | GGDEF |  |
| Sputcn32_1412 | GGDEF | PAS |
| Sputcn32_1741 | GGDEF |  |
| Sputcn32_1800 | GGDEF-EAL | GAF-PAC |
| Sputcn32_1851 | GGDEF-EAL |  |
| Sputcn32_1858 | GGDEF-EAL | GAF-PAC-PAS |
| Sputcn32_1917 | GGDEF-EAL | PAS-PAC |
| Sputcn32_1934 | GGDEF | PBPb |
| Sputcn32_1988 | HD-GYP | GAF |
| Sputcn32_2096 | GGDEF |  |
| Sputcn32_2106 | EAL |  |
| Sputcn32_2362 | HD-GYP |  |
| Sputcn32_2456 | GGDEF-EAL | PAS-PAC |
| Sputcn32_2671 | GGDEF | HAMP |
| Sputcn32_2800 | GGDEF-EAL |  |
| Sputcn32_2830 | GGDEF-EAL | HAMP |
| Sputcn32_3018 | GGDEF | GAF-PAC-PAS |
| Sputcn32_3085 | GGDEF | GAF |
| Sputcn32_3141 | HD-GYP | REC |
| Sputcn32_3168 | GGDEF |  |
| DosD | GGDEF | Globin |
| Sputcn32_3269 | GGDEF | PAS |
| Sputcn32_3306 | GGDEF | PAS |
| Sputcn32_3319 | GGDEF-EAL | CBS |
| Sputcn32_3328 | GGDEF-EAL | REC |
| Sputcn32_3390 | GGDEF | PAS-PAC |
| PdeB | GGDEF-EAL | HAMP-PAS |
| Sputcn32_3598 | GGDEF-EAL | GAF |
| Sputcn32_3648 | GGDEF-EAL | PAS-PAC |
| Sputcn32_3856 | GGDEF-EAL | CHASE-PAS-PAC |
| Sputcn32_3917 | EAL | PAS-PAC |

**Table S2. Strains and plasmids used in this study**

| Strains or Plasmids | Usages or Descriptions | Sources or references |
| --- | --- | --- |
| *Shewanella putrefaciens* CN32 | | |
| Wild type (WT) | *Shewanella putrefaciens* CN32 | (1) |
| Δ*lrbR* | CN32Δ*Sputcn32_0305* | (1) |
| Δ*0099* | CN32 Δ*Sputcn32_0099* | (2) |
| Δ*0133* | CN32 Δ*Sputcn32_0133* | (3) |
| Δ*0327* | CN32 Δ*Sputcn32_0327* | (2) |
| Δ*0384* | CN32 Δ*Sputcn32_0384* | (2) |
| Δ*0414* | CN32 Δ*Sputcn32_0414* | (2) |
| Δ*0555* | CN32 Δ*Sputcn32_0555* | (2) |
| Δ*0601* | CN32 Δ*Sputcn32_0601* | (2) |
| Δ*0654* | CN32 Δ*Sputcn32_0654* | (3) |
| Δ*1039* | CN32 Δ*Sputcn32_1039* | (2) |
| Δ*1235* | CN32 Δ*Sputcn32_1235* | (2) |
| Δ*1253* | CN32 Δ*Sputcn32_1253* | (2) |
| Δ*1291* | CN32 Δ*Sputcn32_1291* | (3) |
| Δ*1365* | CN32 Δ*Sputcn32_1365* | (3) |
| Δ*1412* | CN32 Δ*Sputcn32_1412* | (3) |
| Δ*1741* | CN32 Δ*Sputcn32_1741* | (2) |
| Δ*1800* | CN32 Δ*Sputcn32_1800* | (2) |
| Δ*1851* | CN32 Δ*Sputcn32_1851* | (2) |
| Δ*1858* | CN32 Δ*Sputcn32_1858* | (3) |
| Δ*1917* | CN32 Δ*Sputcn32_1917* | (2) |
| Δ*1934* | CN32 Δ*Sputcn32_1934* | (3) |
| Δ*2096* | CN32 Δ*Sputcn32_2096* | (2) |
| Δ*2106* | CN32 Δ*Sputcn32_2106* | (2) |
| Δ*2456* | CN32 Δ*Sputcn32_2456* | (2) |
| Δ*2671* | CN32 Δ*Sputcn32_2671* | (2) |
| Δ*2800* | CN32 Δ*Sputcn32_2800* | (2) |
| Δ*2830* | CN32 Δ*Sputcn32_2830* | (2) |
| Δ*3018* | CN32 Δ*Sputcn32_3018* | (2) |
| Δ*3085* | CN32 Δ*Sputcn32_3085* | (2) |
| Δ*3168* | CN32 Δ*Sputcn32_3168* | (2) |
| Δ*dosD* | CN32 Δ*Sputcn32_3244* | (4) |
| Δ*3269* | CN32 Δ*Sputcn32_3269* | (2) |
| Δ*3306* | CN32 Δ*Sputcn32_3306* | (2) |
| Δ*3319* | CN32 Δ*Sputcn32_3319* | (3) |
| Δ*3328* | CN32 Δ*Sputcn32_3328* | (3) |
| Δ*3390* | CN32 Δ*Sputcn32_3390* | (2) |
| Δ*pdeB* | CN32 Δ*Sputcn32_3405* | (5) |
| Δ*3598* | CN32 Δ*Sputcn32_3598* | (3) |
| Δ*3648* | CN32 Δ*Sputcn32_3648* | (2) |
| Δ*3856* | CN32 Δ*Sputcn32_3856* | (2) |
| Δ*3917* | CN32 Δ*Sputcn32_3917* | (2) |
| Δ*0814* | CN32 Δ*Sputcn32_0814* | (2) |
| Δ*3141* | CN32 Δ*Sputcn32_3141* | (2) |
| Δ*1988* | CN32 Δ*Sputcn32_1988* | (2) |
| Δ*1344* | CN32 Δ*Sputcn32_1344* | (2) |
| Δ*2362* | CN32 Δ*Sputcn32_2362* | (2) |
| WT/P*_aacC1_*-*bpfA*/BpfA-Flag | WT replacing the promoter region of *bpfA* operon with *aacC1* promoter and BpfA with 3×Flag inserted after residue 3700 aa (11100 bp) in the full-length protein of 4220 aa | (3) |
| WT/P*_aacC1_*-*bpfA*/BpfD*-*FLAG/BpfG-HA | WT replacing the promoter region of *bpfA* operon with *aacC1* promoter, and with a C-terminal 3×Flag-tagged BpfD, and BpfG with 1×HA inserted after residue 221 aa (663 bp) in the full-length protein of 235 aa transformant | (3) |

**Table S3. β-lactam antibiotics used in this research**

| Reagent | Source | Identifier |
| --- | --- | --- |
| Ampicillin | Macklin | A830931-5g |
| Amoxicillin | Macklin | A822839-1g |
| Penicillin G | Macklin | G768670-25g |
| Cloxacillin | Macklin | C970443-250mg |
| Methicillin | Yuanye Bio-Technology | S82852-50mg |
| Carbenicillin | Macklin | C805408-1g |
| Ceftriaxone | Yuanye Bio-Technology | B24430-200mg |
| Ceftazidime | Macklin | C964537-250mg |
| Imipenem | Macklin | R843845-250mg |
| Meropenem | Macklin | M843917-1g |
| Aztreonam | Macklin | A801653-1g |

**Table S4. Primers used in this study**

| Primer | Sequence (5' to 3') | Target gene | |
| --- | --- | --- | --- |
| *lrbR*-QF | TGATTTTGGTGCAGCCCATTCCTC | *lrbR* qRT-PCR |  |
| *lrbR*-QR | CGCAGCTTGACATCTTGGTTCAAT | *lrbR* qRT-PCR |  |
| *Sputcn32_0099*-QF | CCTGTTGGCTATGGTTGTCGCAGC | *Sputcn32_0099* qRT-PCR |  |
| *Sputcn32_0099*-QR | AAGTGCCATCAGAGGTAGGGTATA | *Sputcn32_0099* qRT-PCR |  |
| *Sputcn32_0133*-QF | ATGGCTACGCGCATTATCGAAACC | *Sputcn32_0133* qRT-PCR |  |
| *Sputcn32_0133*-QR | CACATCAAGGCCATCCTCTGGGTA | *Sputcn32_0133* qRT-PCR |  |
| *Sputcn32_0327*-QF | GCCGAATTGTGGTTGATGGGGAAA | *Sputcn32_0327* qRT-PCR |  |
| *Sputcn32_0327*-QR | TCAGCGTATCATACATGGCCCGAG | *Sputcn32_0327 qRT-PCR* |  |
| *Sputcn32_0384*-QF | CGATTCGCTCAGCCAACAAATTGA | *Sputcn32_0384* qRT-PCR |  |
| *Sputcn32_0384*-QR | ATCGGTCGTAACAAGTCCTGCTGA | *Sputcn32_0384* qRT-PCR |  |
| *Sputcn32_0414*-QF | GAGACGCCTGCAACTTATGCAATC | *Sputcn32_0414* qRT-PCR |  |
| *Sputcn32_0414*-QR | AAACGCGTCGTGAGCTTGTCATTC | *Sputcn32_0414* qRT-PCR |  |
| *Sputcn32_0498*-QF | CTCGTCGCAACGATTAACGGCTTC | *Sputcn32_0498* qRT-PCR |  |
| *Sputcn32_0498*-QR | ATCAACGCGAATGCCATGACTACA | *Sputcn32_0498* qRT-PCR |  |
| *Sputcn32_0555*-QF | AGTGTGTTTAAGCCAGCCGTTAAC | *Sputcn32_0555* qRT-PCR |  |
| *Sputcn32_0555*-QR | CGATCAACTGCGTTGCGTTGTCGC | *Sputcn32_0555* qRT-PCR |  |
| *Sputcn32_0601*-QF | GCAGTGGCTTTGCCGATTTAAGCT | *Sputcn32_0601* qRT-PCR |  |
| *Sputcn32_0601*-QR | TATTTCATCCACGGCTAACTGCTT | *Sputcn32_0601* qRT-PCR |  |
| *Sputcn32_0654*-QF | TGGCAGCAGTTGGATCTTGTTTCC | *Sputcn32_0654* qRT-PCR |  |
| *Sputcn32_0654*-QR | AAGCGCATTTCAAGCCAGTTCATA | *Sputcn32_0654* qRT-PCR |  |
| *Sputcn32_0814*-QF | CTGCCTATGACCTTTCGCCTAAGC | *Sputcn32_0814* qRT-PCR |  |
| *Sputcn32_0814*-QR | CACCGTATAGCTTGTCGTGGCATA | *Sputcn32_0814* qRT-PCR |  |
| *Sputcn32_1039*-QF | ACGGAGCTCTGTTTAGGCTTTAGT | *Sputcn32_1039* qRT-PCR |  |
| *Sputcn32_1039*-QR | CATACATTGCATGATCGGCCTGTT | *Sputcn32_1039* qRT-PCR |  |
| *Sputcn32_1235*-QF | TGAGCCTCGATCCCTTGTTGCACC | *Sputcn32_1235* qRT-PCR |  |
| *Sputcn32_1235*-QR | CCAAACTGGTCGCCTGCTTATCTG | *Sputcn32_1235* qRT-PCR |  |
| *Sputcn32_1253*-QF | TACACTACCGCTGCCTGTTGCATT | *Sputcn32_1253* qRT-PCR |  |
| *Sputcn32_1253*-QR | CTCAAGCTCAAAGCGTCTGGACAA | *Sputcn32_1253* qRT-PCR |  |
| *Sputcn32_1291*-QF | AGCGGATCGATGAGGCATTACCAG | *Sputcn32_1291* qRT-PCR |  |
| *Sputcn32_1291*-QR | GCTAATCCCGCTCGACTTTCTTCG | *Sputcn32_1291* qRT-PCR |  |
| *Sputcn32_1344*-QF | GGTACTGCTGCTGAACGTGACTAT | *Sputcn32_1344* qRT-PCR |  |
| *Sputcn32_1344*-QR | TCGTGCGTCTTCTATTGTGGCAAC | *Sputcn32_1344* qRT-PCR |  |
| *Sputcn32_1365*-QF | TTACTCACGCCACTGACACCGTCA | *Sputcn32_1365* qRT-PCR |  |
| *Sputcn32_1365*-QR | CAAACATCGCCTGCGTTGACATTT | *Sputcn32_1365* qRT-PCR |  |
| *Sputcn32_1412*-QF | ATTCCGAGCCATGAGCCATTGTTC | *Sputcn32_1412* qRT-PCR |  |
| *Sputcn32_1412*-QR | GGTTCGCAAGTCCCGTTAGTGAGT | *Sputcn32_1412* qRT-PCR |  |
| *Sputcn32_1741*-QF | GATCAGGTATTGCGTGCCGTTGCT | *Sputcn32_1741* qRT-PCR |  |
| *Sputcn32_1741*-QR | TTAGGCACGAGAATGGCGAACTCT | *Sputcn32_1741* qRT-PCR |  |
| *Sputcn32_1800*-QF | CCGATGTGCGATGCAAACGATTAA | *Sputcn32_1800* qRT-PCR |  |
| *Sputcn32_1800*-QR | ATGCATCCTGCAACTCATGGGACC | *Sputcn32_1800* qRT-PCR |  |
| *Sputcn32_1851*-QF | TTGCTAGTCGACCACGCTTCACAA | *Sputcn32_1851* qRT-PCR |  |
| *Sputcn32_1851*-QR | AGAACGTCCGTGGTGTTGATTGAT | *Sputcn32_1851* qRT-PCR |  |
| *Sputcn32_1858*-QF | TGGCCGATCAGCTTGCATTAACCC | *Sputcn32_1858* qRT-PCR |  |
| *Sputcn32_1858*-QR | CTGTTGCACTGCGAAATAGGCTTA | *Sputcn32_1858* qRT-PCR |  |
| *Sputcn32_1917*-QF | CAGGAGAAGTGATCGGAGCCGAAG | *Sputcn32_1917* qRT-PCR |  |
| *Sputcn32_1917*-QR | CACCCAGCGGGATTTCAAGTTCAC | *Sputcn32_1917* qRT-PCR |  |
| *Sputcn32_1934*-QF | GTTTATCCAGCAGACTGAACGGCG | *Sputcn32_1934* qRT-PCR |  |
| *Sputcn32_1934*-QR | GGGTAAGCCATCAACACCGAGATC | *Sputcn32_1934* qRT-PCR |  |
| *Sputcn32_1988*-QF | GTAGAGCATCACCTATGCACGATA | *Sputcn32_1988* qRT-PCR |  |
| *Sputcn32_1988*-QR | GCTGACGCATGATTTGCCATTCAT | *Sputcn32_1988* qRT-PCR |  |
| *Sputcn32_2096*-QF | GAGGGTGATGATGCCGAAACGCTC | *Sputcn32_2096* qRT-PCR |  |
| *Sputcn32_2096*-QR | CCTAACACAGTTACGCCCATCGGA | *Sputcn32_2096* qRT-PCR |  |
| *Sputcn32_2106*-QF | CCGATGGTAAGGCCATTGCGCCTA | *Sputcn32_2106* qRT-PCR |  |
| *Sputcn32_2106*-QR | GCGGAGCATGAGCGAGCTGAAATG | *Sputcn32_2106* qRT-PCR |  |
| *Sputcn32_2362*-QF | TGGAGCAGCCTTTCTTGACGTTGG | *Sputcn32_2362* qRT-PCR |  |
| *Sputcn32_2362*-QR | TCATCATTGCAATCACCGCAACAG | *Sputcn32_2362* qRT-PCR |  |
| *Sputcn32_2456*-QF | AATGACTCATTGGGCCACCAAGTG | *Sputcn32_2456* qRT-PCR |  |
| *Sputcn32_2456*-QR | CGATTGCAAACTCATCGCCACCTA | *Sputcn32_2456* qRT-PCR |  |
| *Sputcn32_2671*-QF | CACGACCAGACTCAACCACTATGA | *Sputcn32_2671* qRT-PCR |  |
| *Sputcn32_2671*-QR | TCCAGCTTGGCGATTGTTCGAGAG | *Sputcn32_2671* qRT-PCR |  |
| *Sputcn32_2800*-QF | GATACGCCAGTGAGTGTTCGTGTG | *Sputcn32_2800* qRT-PCR |  |
| *Sputcn32_2800*-QR | GGCAAGATCCAGCCTTCGTAACAT | *Sputcn32_2800* qRT-PCR |  |
| *Sputcn32_2830*-QF | TACTATCCAACGGTCGCGTGTACA | *Sputcn32_2830* qRT-PCR |  |
| *Sputcn32_2830*-QR | CCGAGTGCATGACCGAGAGAGTCA | *Sputcn32_2830* qRT-PCR |  |
| *Sputcn32_3018*-QF | GATTTCCTGTGCAGATACGGCTAT | *Sputcn32_3018* qRT-PCR |  |
| *Sputcn32_3018*-QR | GTAGCTCGAGCGGCAGTATCGTAT | *Sputcn32_3018* qRT-PCR |  |
| *Sputcn32_3085*-QF | CACATCAGGCCATAGCGAGTTGTC | *Sputcn32_3085* qRT-PCR |  |
| *Sputcn32_3085*-QR | ACCTGATCGCCCTTACGATGACCA | *Sputcn32_3085* qRT-PCR |  |
| *Sputcn32_3141*-QF | AACAGCCTTGGACCATAGAGGCAA | *Sputcn32_3141* qRT-PCR |  |
| *Sputcn32_3141*-QR | AATGCCTCCACCAGCTTAGGGTCA | *Sputcn32_3141* qRT-PCR |  |
| *Sputcn32_3168*-QF | CCATATCAATGTGCTCGGTGCGTG | *Sputcn32_3168* qRT-PCR |  |
| *Sputcn32_3168*-QR | AGCCACATTACGTAGCGCAGTAAT | *Sputcn32_3168* qRT-PCR |  |
| *dosD*-QF | CGCTCAGAGGAAGCCTATCGCTTA | *Sputcn32_3244* qRT-PCR |  |
| *dosD*-QR | TTCCCAATCTAGGAACGCGGCACG | *Sputcn32_3244* qRT-PCR |  |
| *Sputcn32_3269*-QF | CGCCATGCATTACATTGCCGACTT | *Sputcn32_3269* qRT-PCR |  |
| *Sputcn32_3269*-QR | CGAAAGCCGACAATATCCAAGGCA | *Sputcn32_3269* qRT-PCR |  |
| *Sputcn32_3306*-QF | AACCGAGCATTCATCAGTTGGGAG | *Sputcn32_3306* qRT-PCR |  |
| *Sputcn32_3306*-QR | GTACATAAAGTCAGCGCGACCAGT | *Sputcn32_3306* qRT-PCR |  |
| *Sputcn32_3319*-QF | AATCATGCCGTTAGCGAGGTCATG | *Sputcn32_3319* qRT-PCR |  |
| *Sputcn32_3319*-QR | TTGCTGTGCCACGGTATTTGCACT | *Sputcn32_3319* qRT-PCR |  |
| *Sputcn32_3328*-QF | TTGGTGCCAGTATTGGCGTTGCTT | *Sputcn32_3328* qRT-PCR |  |
| *Sputcn32_3328*-QR | CTTGGCGCGGTACATCGCAATATC | *Sputcn32_3328* qRT-PCR |  |
| *Sputcn32_3390*-QF | CAGGCAGTGTACCGAATGTTGCAT | *Sputcn32_3390* qRT-PCR |  |
| *Sputcn32_3390*-QR | ATCGGCTAAGCGCTCAACAATTCG | *Sputcn32_3390* qRT-PCR |  |
| *pdeB*-QF | AACGGATGGAGATCCTACTGCGAA | *Sputcn32_3405* qRT-PCR |  |
| *pdeB*-QR | ATCAGCTTAAATCGCTCTGCGGCA | *Sputcn32_3405* qRT-PCR |  |
| *Sputcn32_3598*-QF | AGAGCAAGCGATACAACAGCGCAC | *Sputcn32_3598* qRT-PCR |  |
| *Sputcn32_3598*-QR | TTGTAGACGTTCAGCTCTGCGCCT | *Sputcn32_3598* qRT-PCR |  |
| *Sputcn32_3648*-QF | CCGCAGAATGTGACTGCCTATCAA | *Sputcn32_3648* qRT-PCR |  |
| *Sputcn32_3648*-QR | ACCCTGCTGGGAGATAAGGTCATA | *Sputcn32_3648* qRT-PCR |  |
| *Sputcn32_3856*-QF | TTGCTTGAATGCTGGAACCCATAG | *Sputcn32_3856* qRT-PCR |  |
| *Sputcn32_3856*-QR | AAGAGTACACTCCTTGGATTGGGG | *Sputcn32_3856* qRT-PCR |  |
| *Sputcn32_3917*-QF | TTGAGAATGCAGGCGGTTGTACAA | *Sputcn32_3917* qRT-PCR |  |
| *Sputcn32_3917*-QR | CATATCCAGTGCAGCCAATGACAC | *Sputcn32_3917* qRT-PCR |  |

**Supplementary references**

1. Liu C, Yang J, Liu L, Li B, Yuan H, Liu W. 2017. Sodium lactate negatively regulates *Shewanella putrefaciens* CN32 biofilm formation via a three component regulatory system (LrbS-LrbALrbR). Appl Environ Microbiol 83:e00712-17.
2. Sun D, Liu X, Zhang Y, Shi R, Ru Y, Zhou X, Chen Y, Yang J, Liu J, Zhu J, Liu C, Liu W. 2025. Local c-di-GMP signaling, triggered by cross-regulation of cAMP-CRP and c-di-GMP, controls biofilm formation under nutrient limitation. Proc Natl Acad Sci U S A doi:doi:10.1073/pnas.2516964122:e2516964122.
3. Liu C, Sun D, Liu J, Chen Y, Zhou X, Ru Y, Zhu J, Liu W. 2022. cAMP and c-di-GMP synergistically support biofilm maintenance through the direct interaction of their effectors. Nat Commun 13:1493.
4. Cheng YY, Wu C, Wu JY, Jia HL, Wang MY, Wang HY, Zou SM, Sun RR, Jia R, Xiao YZ, Schottel JL. 2017. FlrA represses transcription of the biofilm-associated *bpfA* operon in *Shewanella putrefaciens*. Appl Environ Microbiol 83:e0241016.
5. Rossmann FM, Rick T, Mrusek D, Sprankel L, Dörrich AK, Leonhard T, Bubendorfer S, Kaever V, Bange G, Thormann KM. 2019. The GGDEF domain of the phosphodiesterase PdeB in *Shewanella putrefaciens* mediates recruitment by the polar landmark protein HubP. J Bacteriol 201:e00534-18.
